# Supplementary material for: Helicobacter pylori FabX contains a [4Fe-4S] cluster essential for unsaturated fatty acid synthesis
Source: Nat Commun. 2021 Nov 26;12:6932. doi: 10.1038/s41467-021-27148-0 (PMC8626469; doi:10.1038/s41467-021-27148-0)
Supplement: Supplementary file 1 — Supplementary Information [file 41467_2021_27148_MOESM1_ESM.pdf]

SUPPLEMENTARY INFORMATION FOR

Helicobacter pylori FabX contains a [4Fe-4S] cluster essential for unsaturated fatty acid synthesis

Jiashen Zhou[1,§], Lin Zhang[1,§], Liping Zeng[2,§], Lu Yu[4,§], Yuanyuan Duan[3,§], Siqi Shen[1,§], Jingyan Hu[1], Pan Zhang[5], Wenyan Song[1], Xiaoxue Ruan[6], Jing Jiang[7], Yinan Zhang[7], Lu Zhou[6], Jia Jia[2], Xudong Hang[2], Changlin Tian[4,8], Houwen Lin[1], Hong-Zhuan Chen[9*], John E. Cronan[10*], Hongkai Bi[2*] and Liang Zhang[1*]

[§] These authors contribute equally to this work.

*Correspondences: Liang Zhang, E-mail: liangzhang2014@sjtu.edu.cn; Hongkai Bi, Email: hkbi@njmu.edu.cn; John E. Cronan, E-mail: jecronan@illinois.edu; Hong-Zhuan Chen, E-mail: hongzhuan_chen@hotmail.com.

This PDF file includes:

Supplementary Table 1 to 3;

Supplementary Figure 1 to Figure 30;

Supplementary References.

Supplementary Table 1. Kinetic characterization of FabX and its mutants in catalyzing decanoyl-ACP, octanoyl-ACP, or decanoyl-CoA.

Enzyme	Substrate	K_m (μM)	K_{cat} (s^{-1})	K_{cat}/K_m ($mM^{-1}s^{-1}$)
FabX	decanoyl-ACP	1.65	0.55	333
FabX	octanoyl-ACP	1.87	0.31	165
FabX R164A	decanoyl-ACP	16.18	0.027	1.6
FabX C304A	decanoyl-ACP	2.19	0.0338	15.4
FabX	decanoyl-CoA	125.40	0.0186	0.149

Supplementary Table 2. Data collection and refinement statistics.

	FabX	FabX-holo-ACP	FabX-octanoyl-ACP
**Data collection**			
Space group	$P2_1$	$P2_1$	$P2_12_12_1$
Cell dimensions			
a, *b*, *c*(Å)	42.829, 65.953, 68.891	51.793, 104.443, 99.576	98.894, 100.133, 105.479
α, β, γ (°)	90.00, 96.31, 90.00	90.00, 99.52, 90.00	90.00, 90.00, 90.00
Wavelength (Å)	0.9785	0.9785	0.9785
Resolution (Å)*	50.0-1.70 (1.76-1.70)	50.0-2.80 (2.90-2.80)	72.6-2.30 (2.35-2.30)
R_{merge} (%)	11.5(56.0)	8.9(53.7)	19.6(139.4)
$I/\sigma I$	15.6(3.9)	15.5(1.8)	10.1(2.2)
Completeness (%)	100.0(99.9)	97.2(88.6)	100.0(100.0)
Redundancy	6.5(5.7)	6.6(5.1)	13.1(13.6)
CC1/2 (%)	98.6(85.5)	98.7(84.7)	99.8(85.4)
**Refinement**			
Resolution (Å)	50-1.70	50-2.80	72.6-2.30
No.reflections	41292	21529	46632
R_{work}/R_{free}	0.145/0.173	0.208/0.243	0.242/0.286
No.atoms			
Protein	2840	6730	6350
Water	494	44	195
Ligand/ion	40	120	150
B-factors			
Protein	15.454	50.067	46.097
Water	28.065	34.833	42.871
Ligand/ion	11.078	42.676	40.612
R.m.s deviations			
Bond lengths (Å)	0.009	0.020	0.013
Bond angles (°)	1.161	1.716	1.871

*Highest-resolution shell is shown in parentheses.

Supplementary Table 3. Bacterial strains, plasmids and oligonucleotides used in this study.

Bacterial Strains	Genotype	References or sources
E. coli		
DH5α	Δ(*argF–lac*)169 φ80d*lacZ58*(M15) *ΔphoA8 glnV44 deoR481 gyrA96, recA1 endA1, hsdR17*	Lab stock
BL21 (Tuner)	F^{-} *ompT* $hsdS_B$ (r_B^{-} m_B^{-}) *gal dcm lacY1*	Novagen
Rosetta™(DE3) pLysS	F^{-} *ompT* $hsdS_B$ (r_B^{-} m_B^{-}) *gal dcm* (DE3) pLysSRARE	Novagen
H. pylori		
G27	Reference strain	Ref. 1
NSH57	a mouse-adapted derivative of the G27 strain	Ref. 1
BHKS485	Cm^r ,G27 IR0203::*fabX*	This study
BHKS486	Cm^r ,G27 IR0203::*fabX R164A*	This study
BHKS487	Km^r Cm^r; G27 IR0203::*fabX* Δ*fabX*	This study
BHKS488	Km^r Cm^r; G27 IR0203::*fabX R164A* Δ*fabX*	This study
BHKS550	Cm^r; NSH57 IR0203::*fabA*	This study
BHKS551	Km^r Cm^r; NSH57 IR0203::*fabA* Δ*fabX*	This study
BHKS567	Cm^r; NSH57 IR0203::*fabX*	This study
BHKS568	Km^r Cm^r; NSH57 IR0203::*fabX* Δ*fabX*	This study
S. pneumonia		
ATCC 49619	Reference strain	American Type Culture Collection
**Plasmids**		
pET28b	Km^r; T7 promoter-based expression vector, pBR322 origin	Novagen
pET16b	Amp^r; expression vector with a T7 promoter	Novagen
pQE-2	Amp^r; expression vector with a T5 promoter	Qiagen
pZL268	Amp^r; *H. pylori* ACP cloned into pET32a	Ref. 2
pYFJ84	Amp^r; *Vibro aasS* cloned into the NdeI and BamHI sites of pET16b	Ref. 3
pBHKP109	Amp^r Km^r; PCR-amplified *aphA3* cloned into pBluescript SK(+)	Ref. 4
pBHKP252	Cm^r; PCR-amplified *Hp0203up*, *catGC* plus *Hp0204dn* cloned into pBHKP202	Ref. 4
pBHK716	Amp^r; PCR-amplified *fabX* cloned into the NdeI and SalI sites of pQE-2	This study
pBHK717	Amp^r, FabX H182F of pBHK716	This study
pBHK718	Amp^r, FabX H182Q of pBHK716	This study

pBHK719	Amp^r, FabX R164A of pBHK716	This study
pBHK720	Amp^r, FabX C300A of pBHK716	This study
pBHK721	Amp^r, FabX C300S of pBHK716	This study
pBHK722	Amp^r, FabX C304A of pBHK716	This study
pBHK723	Amp^r, FabX C304S of pBHK716	This study
pBHK724	Amp^r, FabX C308A of pBHK716	This study
pBHK725	Amp^r, FabX C308S of pBHK716	This study
pBHK726	Amp^r, FabX C320A of pBHK716	This study
pBHK727	Amp^r, FabX C320S of pBHK716	This study
pBHKP405	Cm^r; PCR-amplified *fabX* containing Pro$_{ureI}$ inserted between the XhoI and SalI sites of pBHKP252	This study
pBHKP406	Cm^r; FabX R164A of pBHK405	This study
pBHKP408	Km^r; PCR-amplified *fabX*up inserted between EcoRI and PstI sites plus PCR-amplified *fabX*dn inserted between BamHI and XbaI sites of pBHKP109	This study
pBHKP421	Cm^r; Synthesized *fabA* (Codon optimized) containing Pro$_{ureI}$ inserted between the XhoI and SalI sites of pBHKP252	This study
pBHKP432	Cm^r; Synthesized *fabX* containing Pro$_{ureI}$ inserted between the XhoI and SalI sites of pBHKP252	This study

**Oligonucleotides**	**Sequence**
P1 (pBS0203Cm04-KpnI-1)	AATTGGTACCGGTTTAGAAGCGCAAAGT
P2 (pBS0203Cm04-XhoI-2)	GTCACTCGAGGCTGTTAATACTAATCCAA
P3 (pBS0203Cm04-BamHI-3)	AGATGGATCCAAGCCAATTCTTCCAATGA
P4 (pBS0203Cm04-XbaI-4)	AATTTCTAGAATGAGCCGGTGGTGATCA
P5 (CatGC-PstI-L)	CTGACTGCAGGATCCGCCATATTGTGTTGA
P6 (CatGC-BamHI-R)	ATATGGATCCCGCAGAACTGGTAGGTATGGA
P7 (HpFabXup-EcoRI)	CTGAGAATTCTATTAGAAGGGCTTGATG
P8 (HpFabXup-PstI)	TAAGCTGCAGATACCATAGCTCTTTCCT
P9 (Km-aphAL-BamHI)	ATAAACTGCAGGCGAACCATTTGAGGTGATAG
P10 (Km-aphAL-PstI)	CGCGCGGATCCTTAAAACAATTCATCCAGTA
P11 (HpFabXDn-R)	CGCATGCTTAGTTGGTGTTTTCTCTT
P12 (FabX R164A-L)	GTAAAAGATGGAGCGATGCCTATAAAAGAATCC
P13 (FabX R164A-R)	CGGGATTCTTTTATAGGCATCGCTCCATCTTTTA
P14 (Promoter-L)	ATCAGAGAGCGATTTTGA
P15 (FabX H182F-L)	GCCTTTGAGTGGGGGGTTTCAGGGCTTTAAATAC
P16 (FabX H182F-R)	GTATTTAAAGCCCTGAAACCCCCCACTCAAAGGC
P17 (FabX H182Q-L)	CTTTGAGTGGGGGGCAGCAGGGCTTTAAATAC

P18 (FabX H182Q-R)	GTATTTAAAGCCCTGCTGCCCCCCACTCAAAG
P19 (FabX C300A-L)	CGCCCAAAATCGCAGCCGTGAGCAATTGTG
P20 (FabX C300A-R)	CACAATTGCTCACGGCTGCGATTTTGGGCG
P21 (FabX C300S-L)	CCCAAAATCGCAAGCGTGAGCAATTG
P22 (FabX C300S-R)	CAATTGCTCACGCTTGCGATTTTGGG
P23 (FabX C304A-L)	GCGTGAGCAATGCGGTAGCGCCTTG
P24 (FabX C304A-R)	CAAGGCGCTACCGCATTGCTCACGC
P25 (FabX C304S-L)	GCGTGAGCAATAGCGTAGCGCCTTG
P26 (FabX C304S-R)	CAAGGCGCTACGCTATTGCTCACGC
P27 (FabX C308A-L)	CAATTGTGTAGCGCCTGCGAACAGGGGTGAAGAG
P28 (FabX C308A-R)	CTCTTCACCCCTGTTCGCAGGCGCTACACAATTG
P29 (FabX C308S-L)	CAATTGTGTAGCGCCTAGCAACAGGGGTGAAG
P30 (FabX C308S-R)	CTTCACCCCTGTTGCTAGGCGCTACACAATTG
P31 (FabX C320A-L)	CTAAAAAGGTGGGCTATGCGATCGCTGATGGTTTGG
P32 (FabX C320A-R)	CCAAACCATCAGCGATCGCATAGCCCACCTTTTTAG
P33 (FabX C320S-L)	CTAAAAAGGTGGGCTATAGCATCGCTGATGGTTTGG
P34 (FabX C320S-R)	CCAAACCATCAGCGATGCTATAGCCCACCTTTTTAG
The DNA sequence of synthesized fragment containing the *ureI* promoter (blue) and *E.coli fabA* (red) optimized by *H. pylori* codons.	CTCGAGCCTTAAATCCTTAGTTTTTAGCTCTCTGATTTTTT<br>GTTTATCAAAAAATTGGGGGCTTTTTTTGTTTTTATTTTTT<br>GTCAATTTACTATTTTTCTTTATGATTAGCTCAAGCAACAA<br>AAGTTATTCGTAAGGTGCGTTTGTTGTAAAAATTTTTGTTT<br>GGAAGGAAAAGGCAATGGTGGATAAAAGGGAAAGCTATA<br>CCAAAGAAGATTTATTAGCTAGCGGGAGGGGGGAATTAT<br>TTGGGGCTAAAGGGCCTCAATTACCTGCTCCTAATATGTT<br>AATGATGGATAGGGTGGTGAAAATGACCGAAACCGGGGG<br>GAATTTTGATAAAGGGTATGTGGAAGCTGAATTAGATATT<br>AATCCTGATTTATGGTTTTTTGGGTGCCATTTTATTGGGGA<br>TCCTGTGATGCCTGGGTGCTTAGGGTTAGATGCTATGTGG<br>CAATTAGTGGGGTTTTATTTAGGGTGGTTAGGGGGGGAAG<br>GGAAAGGGAGGGCTTTAGGGGTGGGGGAAGTGAAATTTA<br>CCGGGCAAGTGTTACCTACCGCTAAAAAAGTGACCTATAG<br>GATTCATTTTAAAAGGATTGTGAATAGGAGGTTAATTATG<br>GGGTTAGCTGATGGGGAAGTGTTAGTGGATGGGAGGTTA<br>ATTTATACCGCTAGCGATTTAAAAGTGGGGTTATTTCAAG<br>ATACCAGCGCTTTTTAAGTCGAC

The underlined sequences are restriction sites.

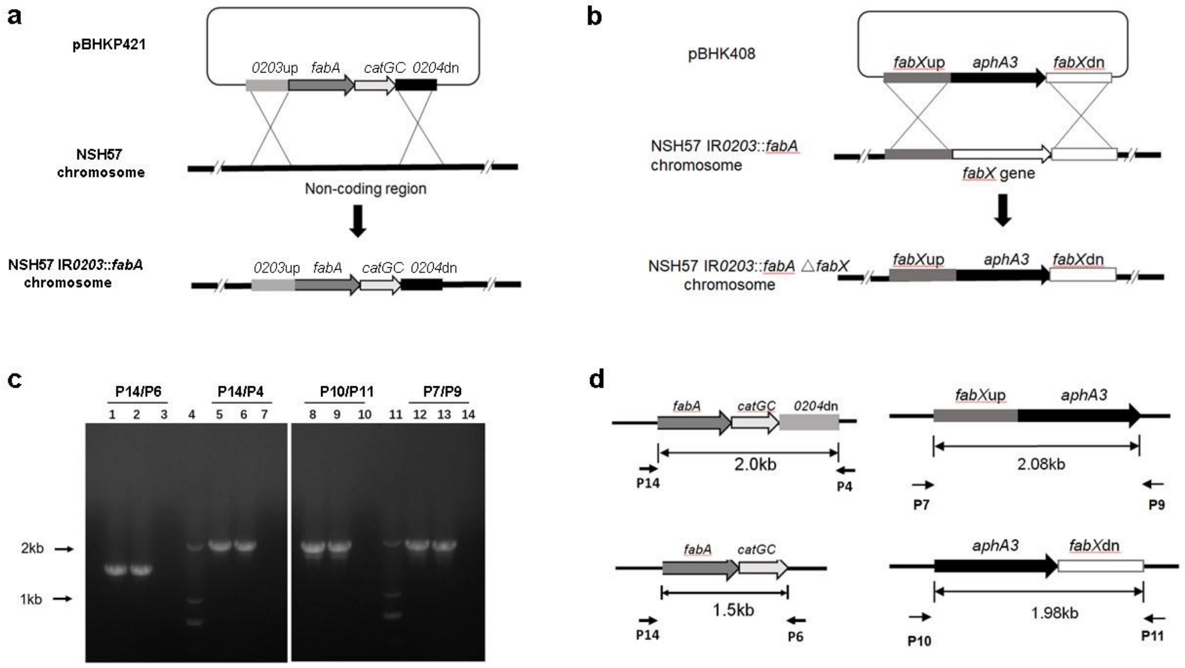

Supplementary Figure 1. Construction and PCR confirmation of strain BHKS551 (NSH57 IR203::*fabA* ∆*fabX*).

(a) Recombination events required to generate the strain using an improved pIR203C04 complementation system by natural transformation. (b) Recombination events required to generate the *fabX* knockout strains using chromosomal complementation system by natural transformation. (c)(d) PCR confirmation of the constructed strain. As shown in c, expected product size using the primer pairs P14/P6 and P14/P4 for *fabA* insertion into the intergenic region is 1.5 and 2.0kb, while the expected product size using the primer pair P7/P9 and P10/P11 for *fabX* knockout is 2.08 and 1.98kb, respectively. From left to right, the temples (lanes 1-3 and lanes 5-7) used in the PCR reactions are the plasmid DNA of pBHKP421, the genomic DNAs of the strain BHKS551 and NSH57, respectively, while the temples (lanes 8-10 and lanes 12-14) are the plasmid DNA of pBHKP408, the genomic DNAs of the strain BHKS551 and NSH57, respectively. The experiment was repeated twice independently with similar results. Source data are provided as a Source Data file.

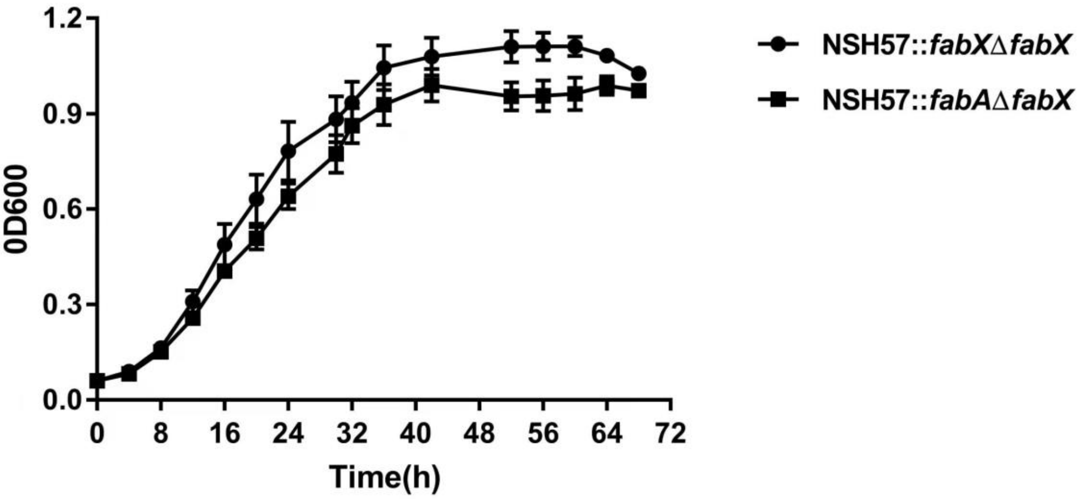

Supplementary Figure 2. The growth phenotypes of the strains BHKS551 (NSH57 IR0203::*fabA* Δ*fabX*) and BHKS568 (NSH57 IR0203::*fabX* Δ*fabX*). The strains were cultured in BHI medium containing 10% FCS. $N = 3$ biologically independent experiments. Data is presented as mean ± SD. Source data are provided as a Source Data file.

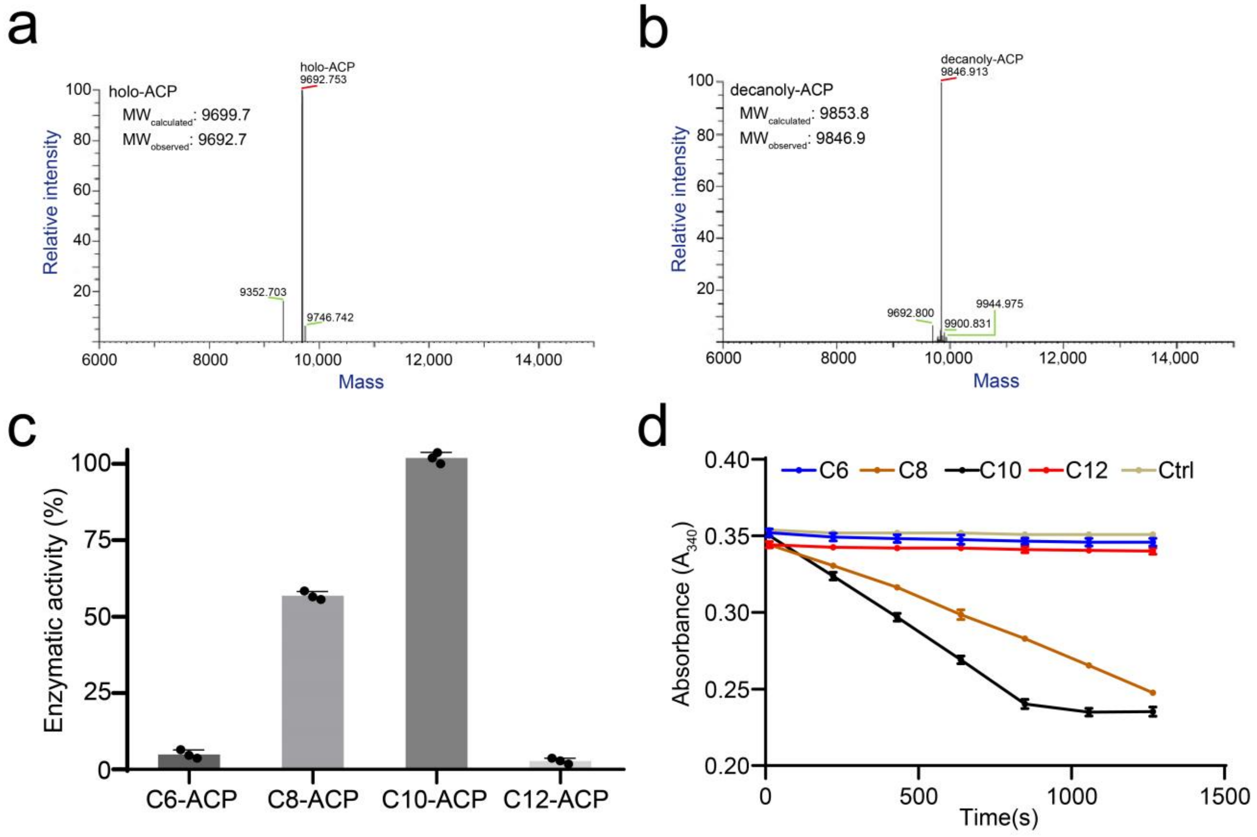

Supplementary Figure 3. Substrate length preference of FabX. (a)(b) Mass spectral analysis of holo-ACP and decanoyl-ACP. The determined molecular wights ($MW_{observed}$) as well as the theoretically calculated molecular wights ($MW_{calculated}$) were shown. (c)(d) Dehydrogenation activity of FabX against various lengths of acyl-ACP substrates (hexanoyl-, octanoyl-, decanoyl-, and dodecanoyl-) determined by the FabX-FabI coupled enzymatic assay. $N = 3$ biologically independent experiments. Data is presented as mean ± SD. Source data are provided as a Source Data file.

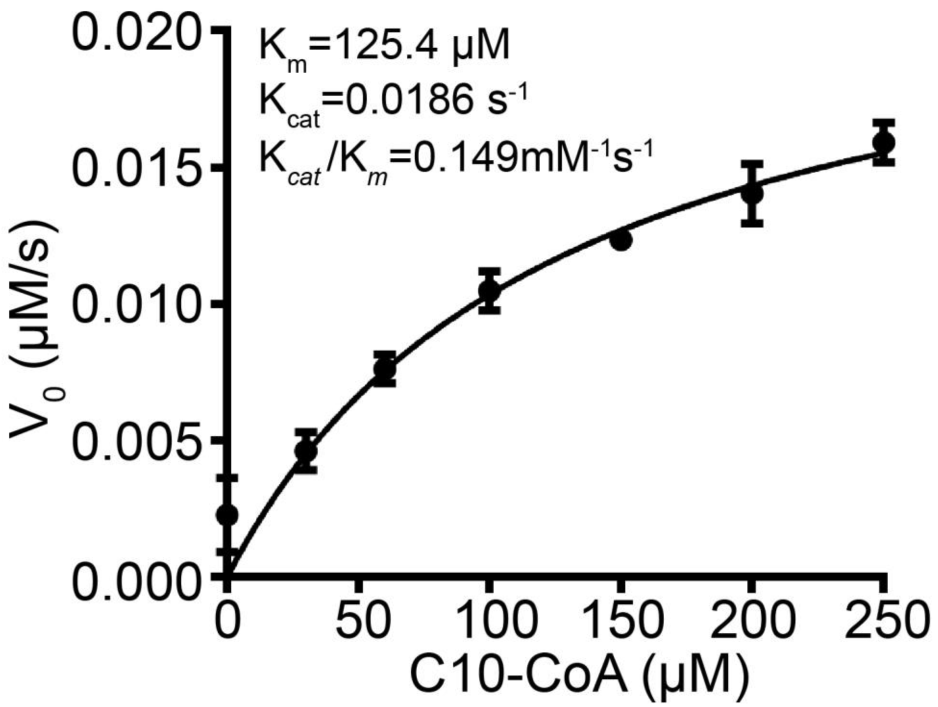

Supplementary Figure 4. Kinetic characterization of FabX in catalyzing dehydrogenation of decanoyl-CoA. The curve was fitted to the Michaelis-Menten equation. $N = 3$ biologically independent experiments. Data is presented as mean ± SD. Source data are provided as a Source Data file.

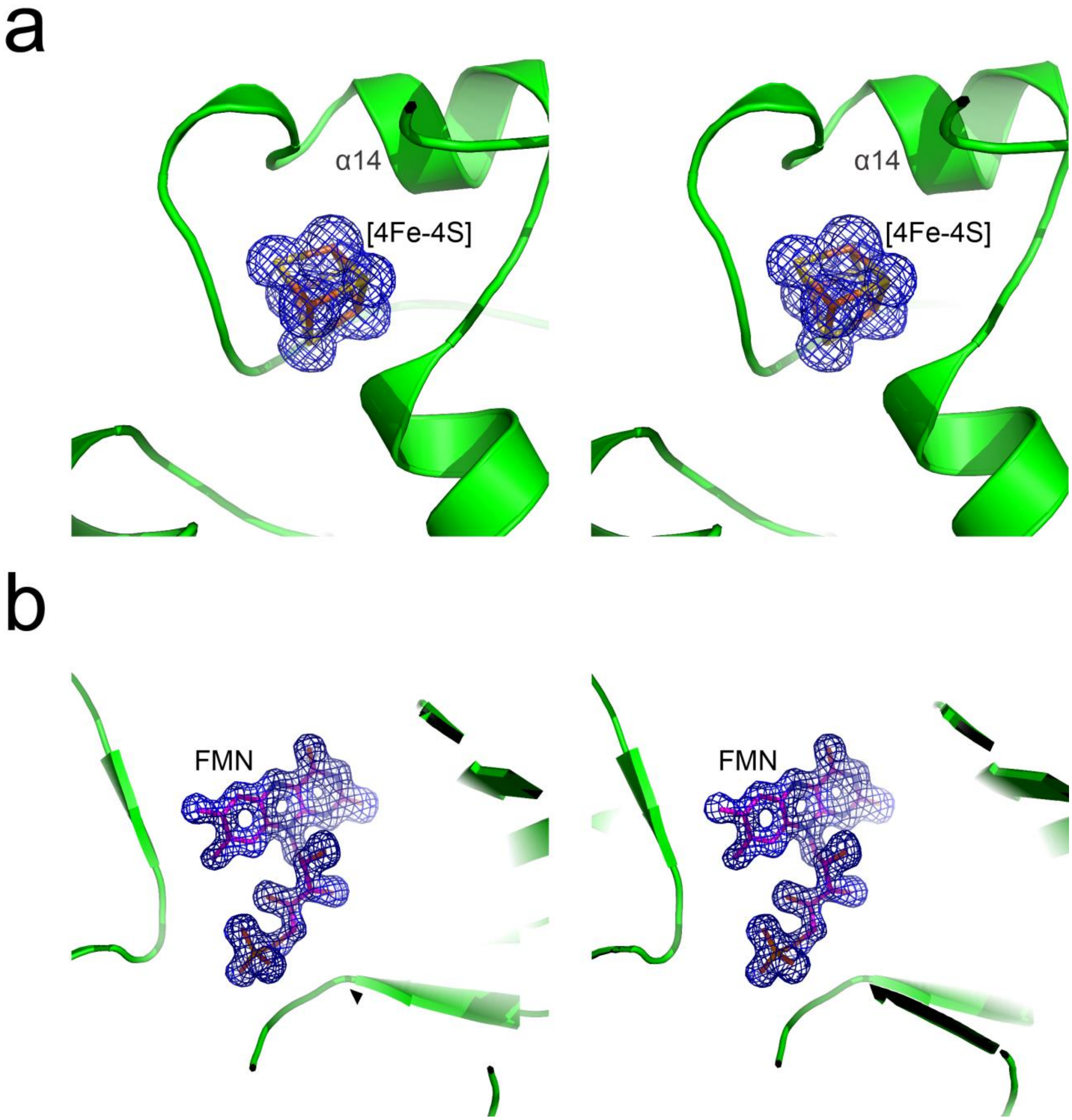

Supplementary Figure 5. Ligand electron density and conformation of FabX. Stereo view of the fofc omit map contoured at 3.0σ around the (a) [4Fe-4S] cluster and (b) FMN cofactor in FabX structure.

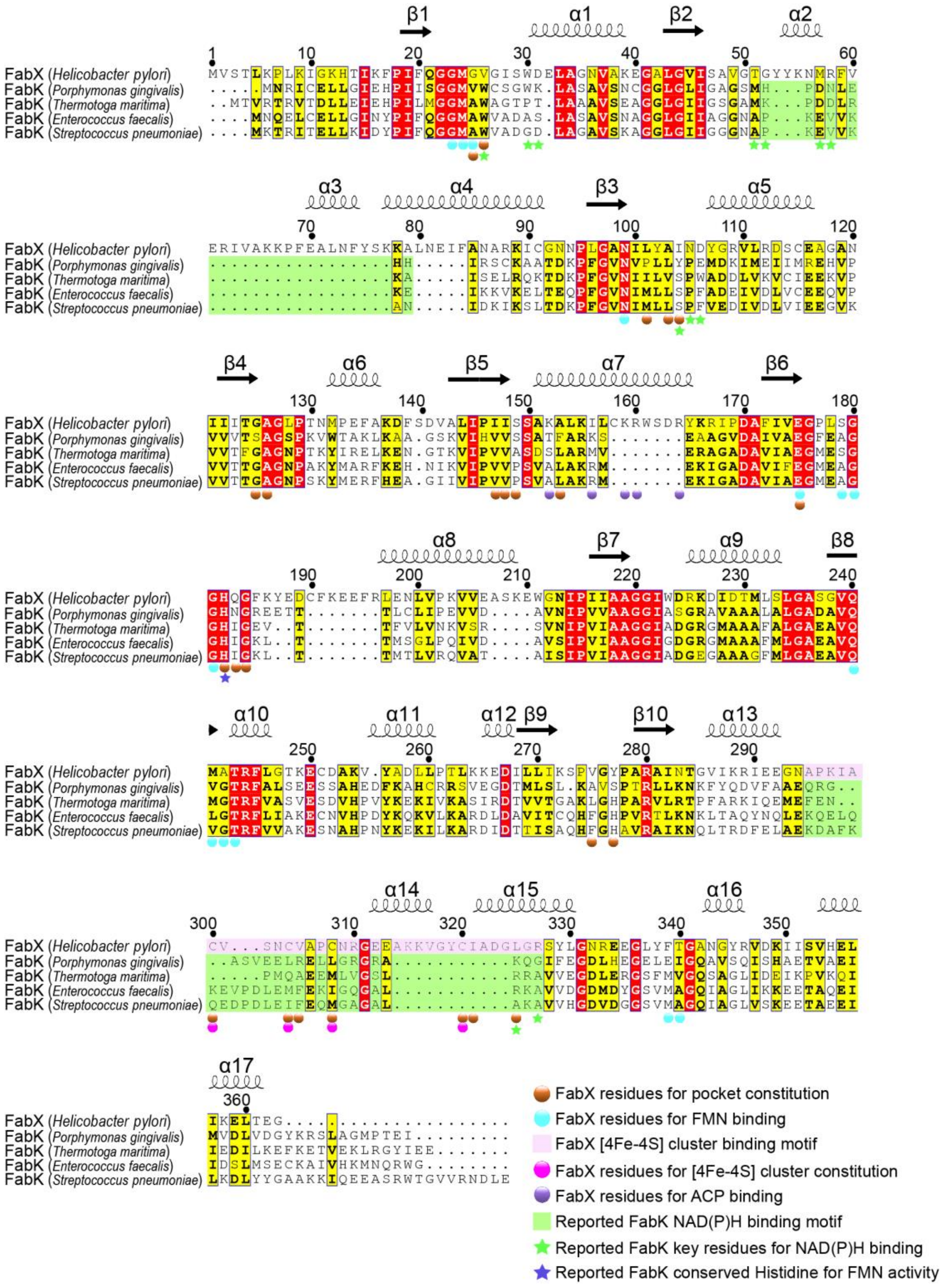

Supplementary Figure 6. Multiple sequence alignment of FabX and FabK family. Secondary structures of FabX were shown and labeled. The [4Fe-4S] motif in FabX and the corresponding NAD(P)H motifs in FabK family were shaded in pink and light green, respectively.

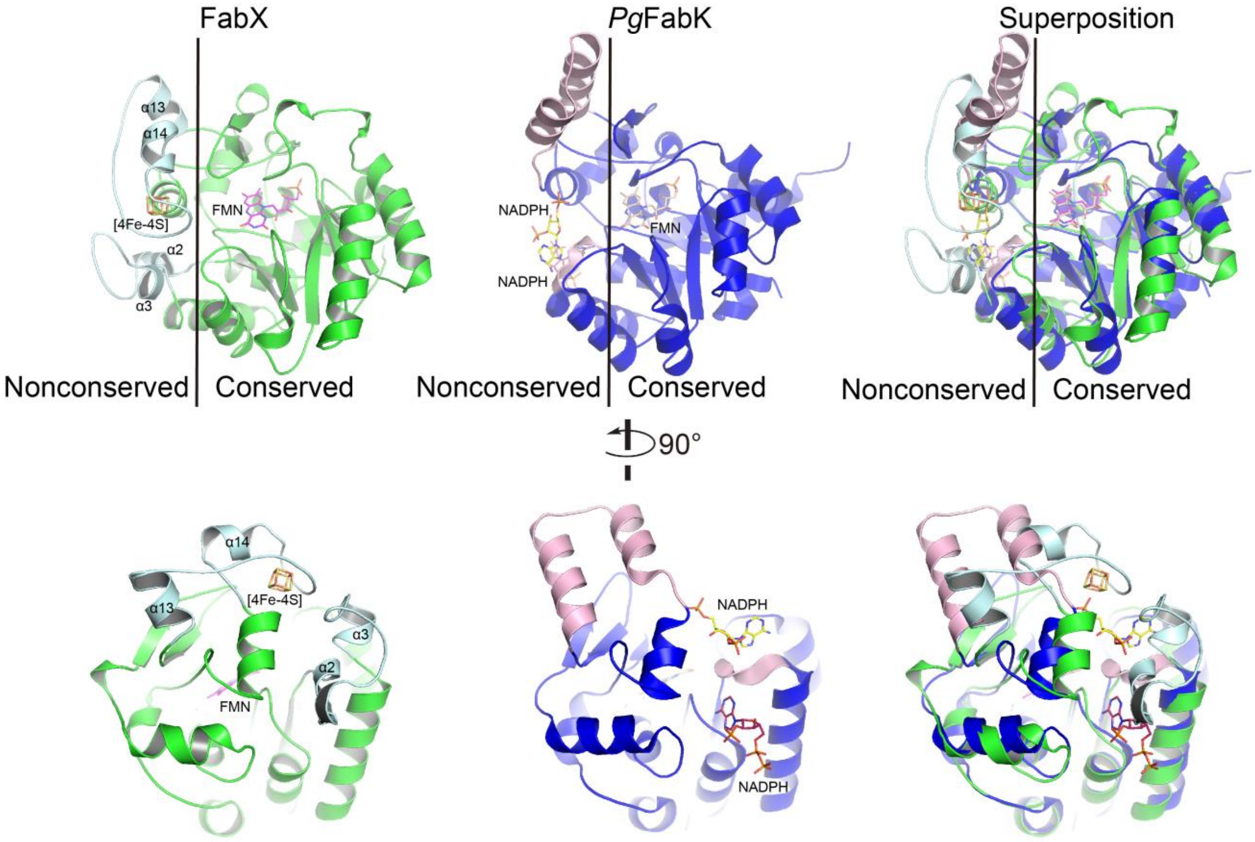

Supplementary Figure 7. Structural superposition of FabX with FabK from *Porphyromonas gingivalis* (*Pg*FabK). FabX, the [4Fe-4S] cluster binding motif of FabX, *Pg*FabK and the NAD(P)H binding motif of *Pg*FabK are colored in green, pale cyan, blue, and light pink, respectively. The FMN cofactor and the [4Fe-4S] cluster of FabX, and the two NADPH ligands of *Pg*FabK were shown in sticks, and colored in magenta, orange, yellow and red, respectively.

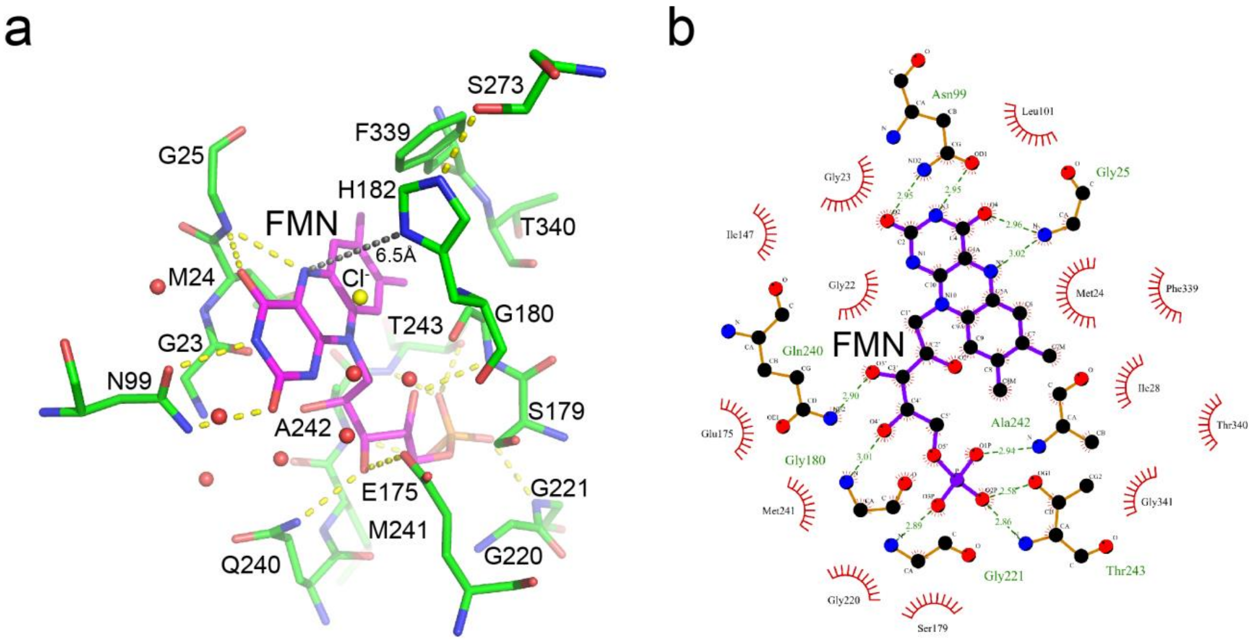

Supplementary Figure 8. The FMN binding motif of FabX. (a) Residues and water molecules that involved in FMN binding were shown in green sticks and red spheres, respectively. The chloride ion locked in the oxyanion hole is shown as a yellow sphere. Yellow dashes indicate hydrogen bonds between FMN and residues. Grey dashes indicate the distance between the sidechain of H182 and FMN. (b) The interactions between FMN and surrounding FabX residues.

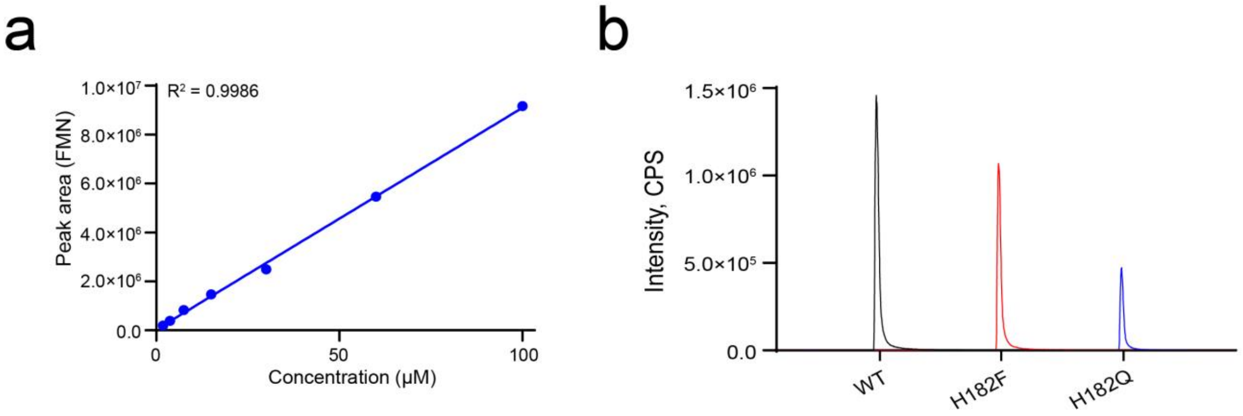

Supplementary Figure 9. Quantitative analysis of FMN contents of FabX and its mutants by LC-MS/MS. (a) Standard curve of FMN. (b) FMN signal from FabX, FabX H182F and FabX H182Q detected by LC-MS/MS. Source data are provided as a Source Data file.

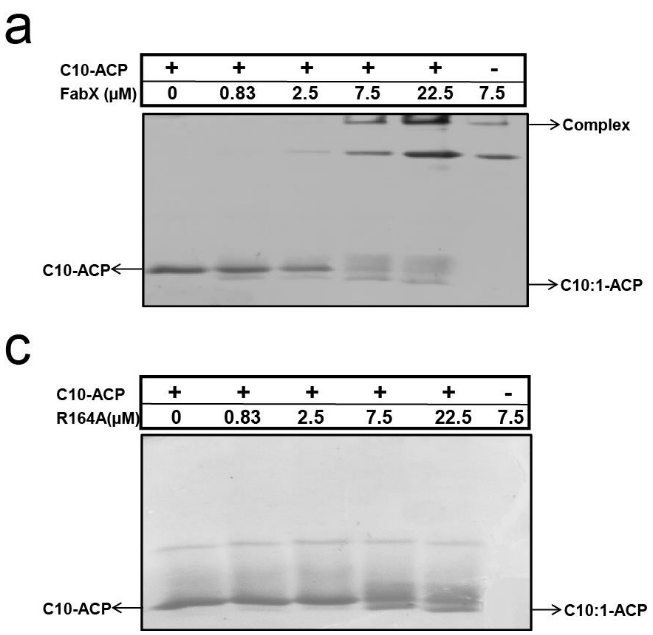

Supplementary Figure 10. The catalytic properties of FabX and its mutants were tested *in vitro* with decanoyl-ACP as the substrate. (a) FabX wild type. (b) FabX H182F. (c) FabX R164A. (d) FabX C300A. The experiment was repeated twice independently with similar results. The reaction mixtures were as described in Experimental Procedures. After incubation at 37°C for 20 min, the reaction products were resolved by conformationally sensitive gel electrophoresis on 18% polyacrylamide gels containing 2.5 M urea[5]. The minus sign denotes a reaction lacking decanoyl-ACP. Source data are provided as a Source Data file.

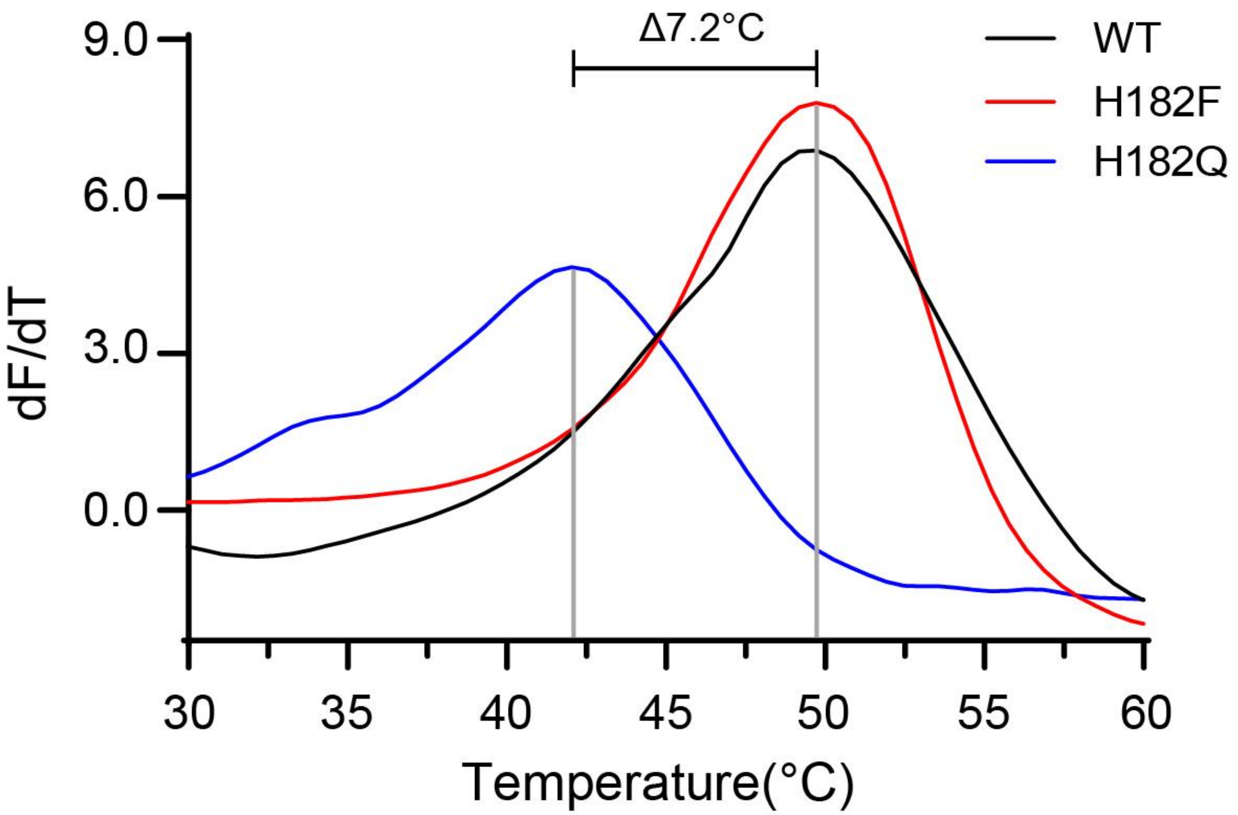

Supplementary Figure 11. Protein stability determinations of FabX, FabX H182F or FabX H182Q by protein thermal shift assay (PTSA). Source data are provided as a Source Data file.

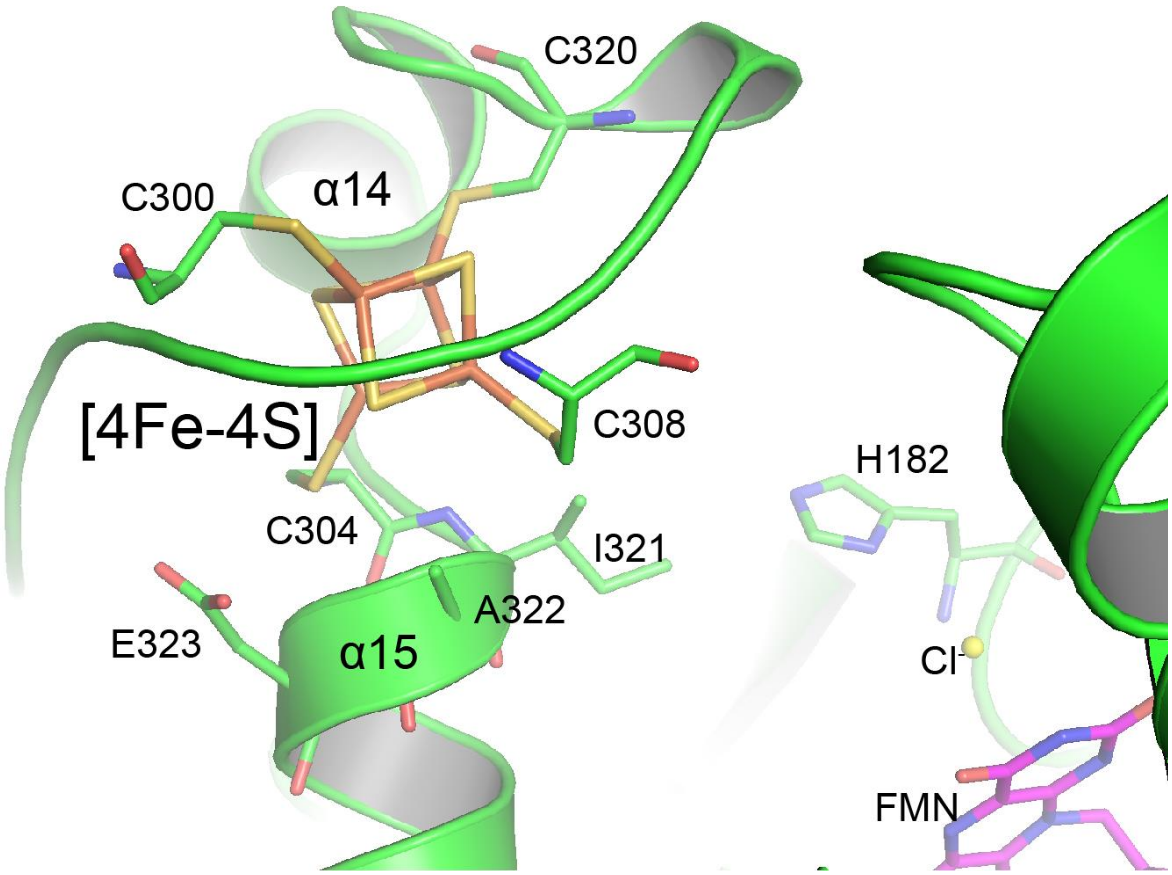

Supplementary Figure 12. Interactions between the [4Fe-4S] cluster and residues located on the N-terminus of helix α15.

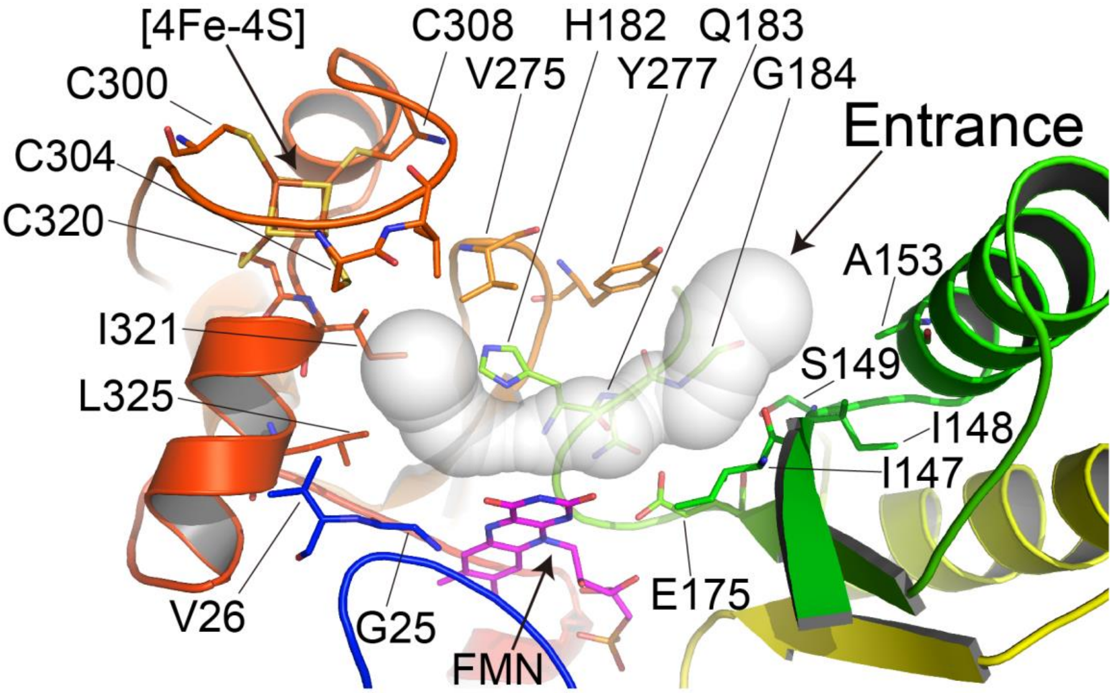

Supplementary Figure 13. The "L" shaped hydrophobic active tunnel of FabX. The white surface of the tunnel was generated by using Pymol plugin Caver 3.0[6]. Residues that constitute the tunnel were shown in sticks and labeled. The entrance of the tunnel, FMN cofactor, and [4Fe-4S] cluster were indicated by black arrows.

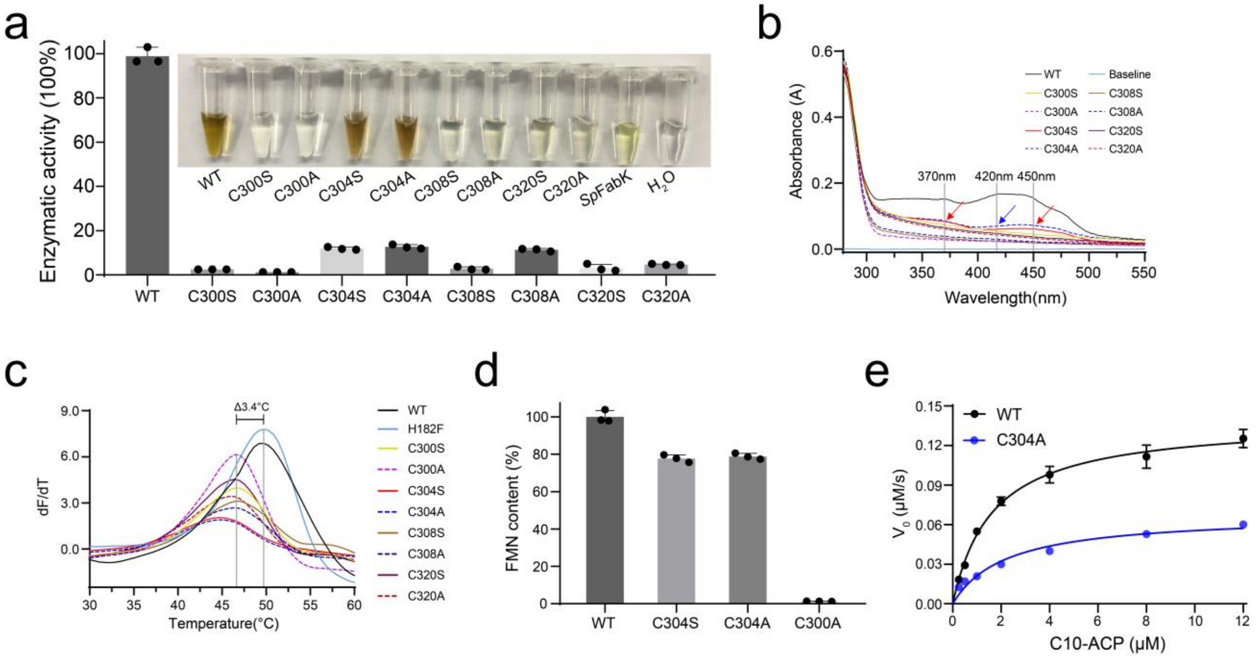

Supplementary Figure 14. Catalytic mechanism of FabX [4Fe-4S] cluster. (a) Dehydrogenase activity of FabX variants. The tubes contain protein solutions of equal concentration and the proteins are labeled. $N = 3$ biologically independent experiments. Data is presented as mean ± SD. (b) Protein stability determinations of FabX variants by Protein Thermal Shift Assay (PTSA). (c) Absorbance screening against FabX variants from 280 to 550 nm. FMN shows the characteristic absorptions at 370 nm and 450 nm (red arrows), while the [4Fe-4S] cluster shows characteristic absorption under 420 nm (blue arrows)[7,8]. (d) The FMN contents of FabX variants were determined by LC-MS/MS. $N = 3$ technical replicates. Data is presented as mean ± SD. (e) Kinetic characterization of FabX C304A in catalyzing the dehydrogenation of decanoyl-ACP. $N = 3$ biologically independent experiments. Data is presented as mean ± SD. Source data are provided as a Source Data file.

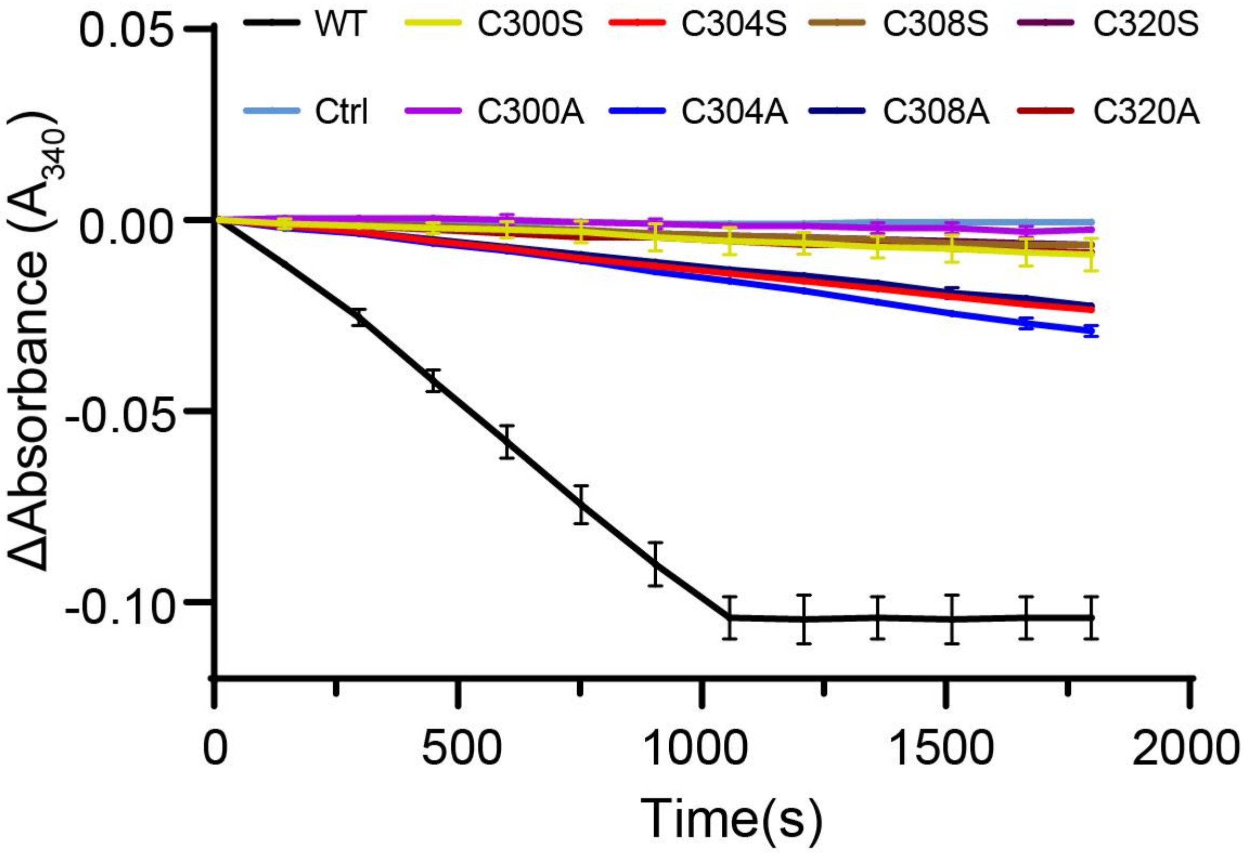

Supplementary Figure 15. Dehydrogenation activities of FabX mutants against decanoyl-ACP determined by the FabX-FabI coupled assay. $N = 3$ biologically independent experiments. Data is presented as mean ± SD. Source data are provided as a Source Data file.

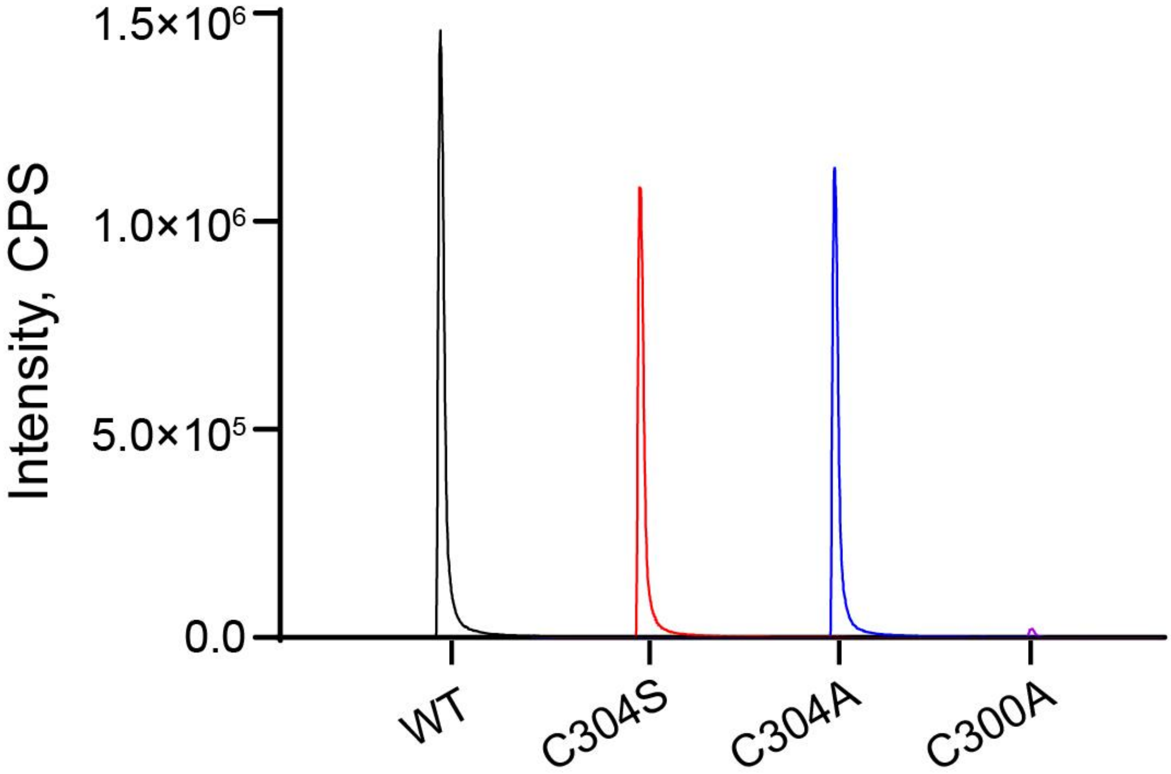

Supplementary Figure 16. Quantitative analysis of FMN content of FabX and its {4Fe-4S} cluster mutants by LC-MS/MS. FMN signal from FabX, C304S, C304A, and C300A detected by LC-MS/MS. Source data are provided as a Source Data file.

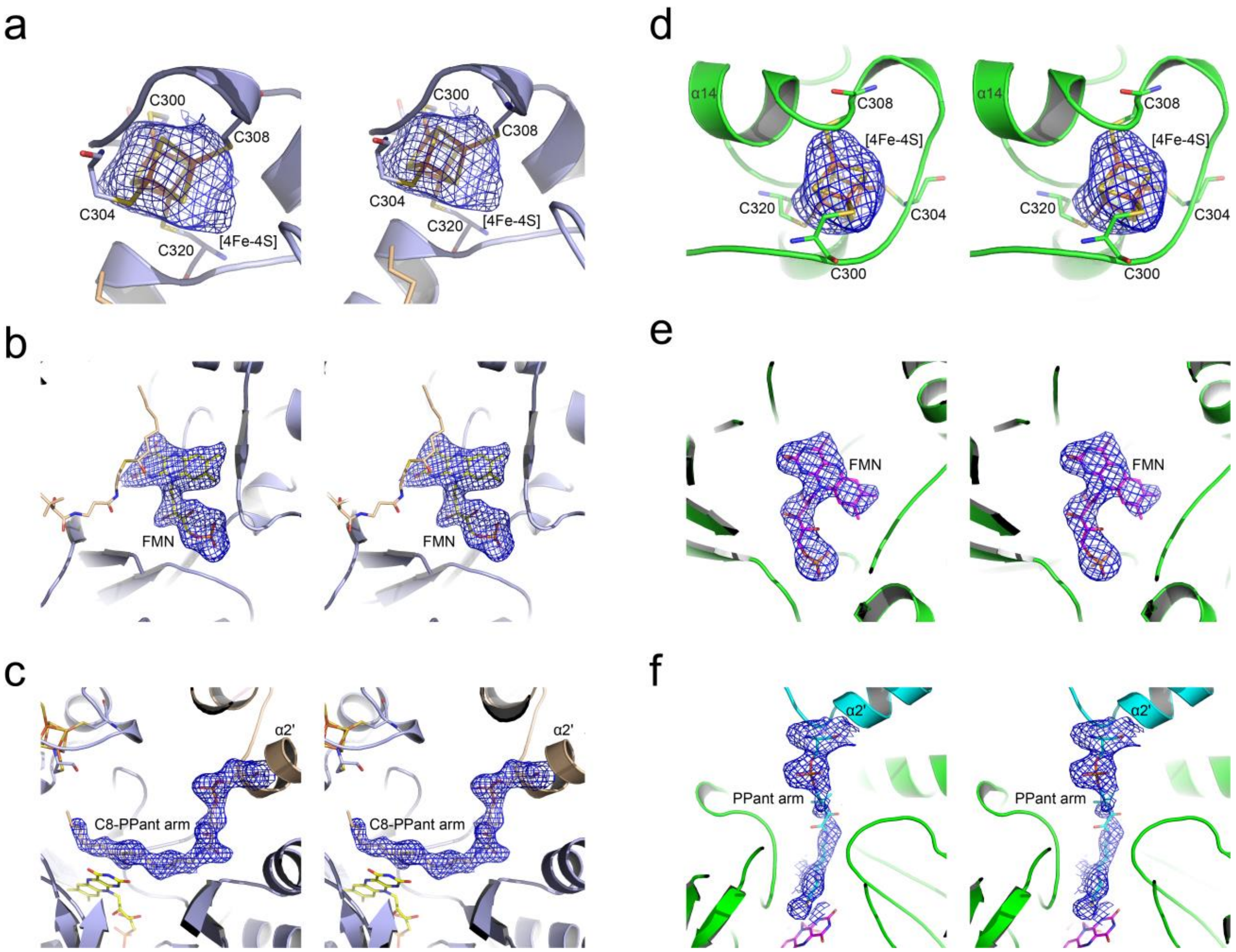

Supplementary Figure 17. Ligand electron density and conformation of FabX-ACP complexs. Stereo view of the fofc omit map contoured at 3.0σ around the (a) (d)[4Fe-4S] cluster, (b) (e) FMN cofactor and the (c) octanoyl-PPant or (f) PPant prosthetic group in FabX-octanoyl-ACP or FabX-holo-ACP complex structures.

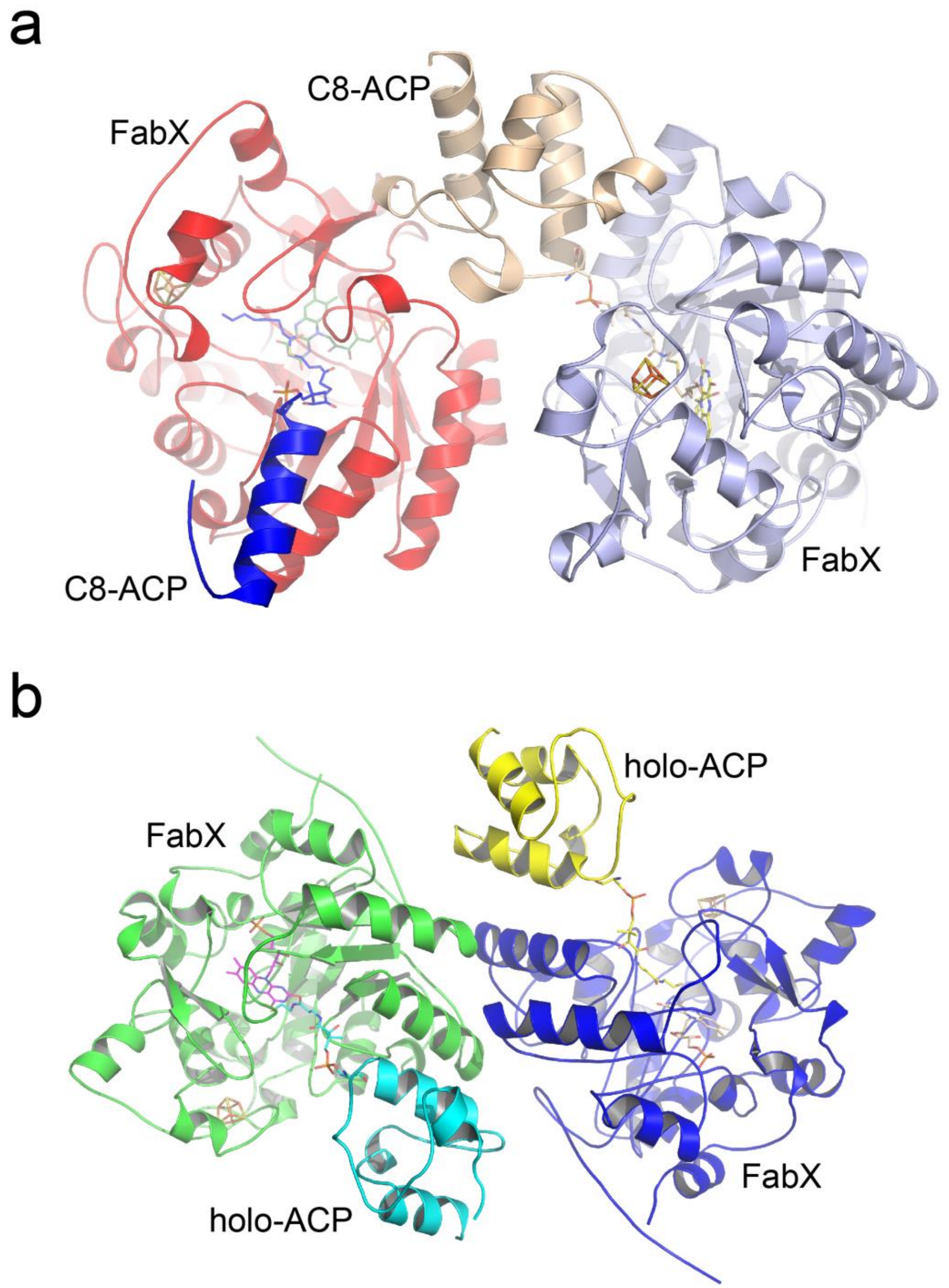

Supplementary Figure 18. The overall crystal structure of FabX in complex with octanoyl-ACP or holo-ACP. (a) The two FabX-octanoyl-ACP complexes are colored in light blue/wheat and red/blue, respectively. One of the complexes (light blue/wheat) is well determined, while the ACP in the other complex (blue) only shows the key α2 helix. The remaing part of it is disordered in the structure due to discontinuous electron density. (b) The two FabX-holo-ACP complexes wae colored in yellow/cyan and blue/yellow, respectively.

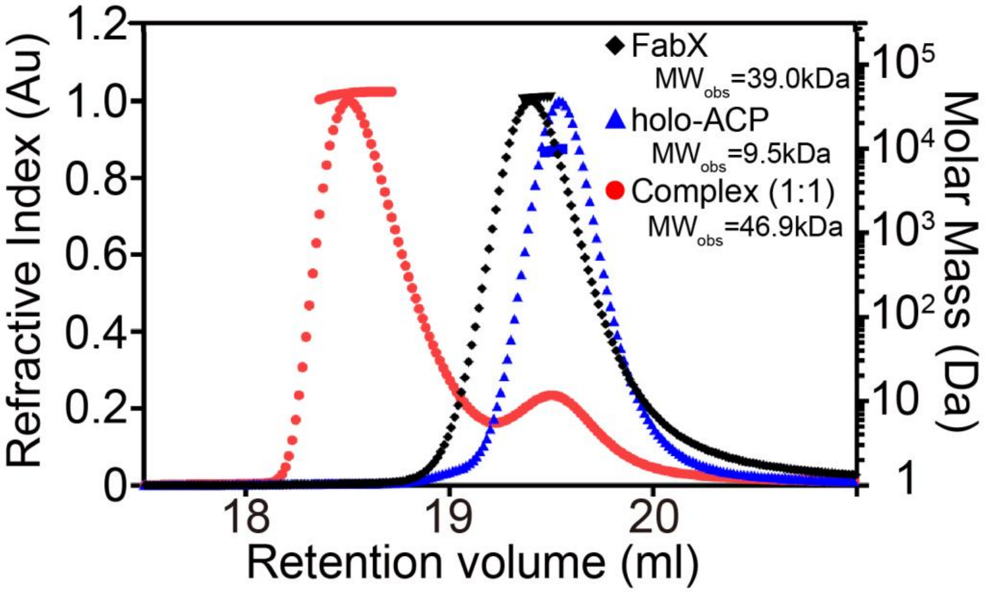

Supplementary Figure 19. Size-exclusion chromatography (SEC) coupled with multi-angle light scattering (MALS) analysis. FabX (black), holo-ACP (blue) and FabX-holo-ACP complex (red) was separated using a Wyatt Technology WTC-030S5 column. The corresponding average molecular weight of the fraction peaks were evaluated and analyzed using a Wyatt Technology SEC-MALS system. The left-hand Y-axes indicate the 280 nm absorption reading scale of the UV detector, while the right-hand Y-axes indicate the molecular mass. The observed molecular wights of FabX, holo-ACP and the complex wae also shown. Source data are provided as a Source Data file.

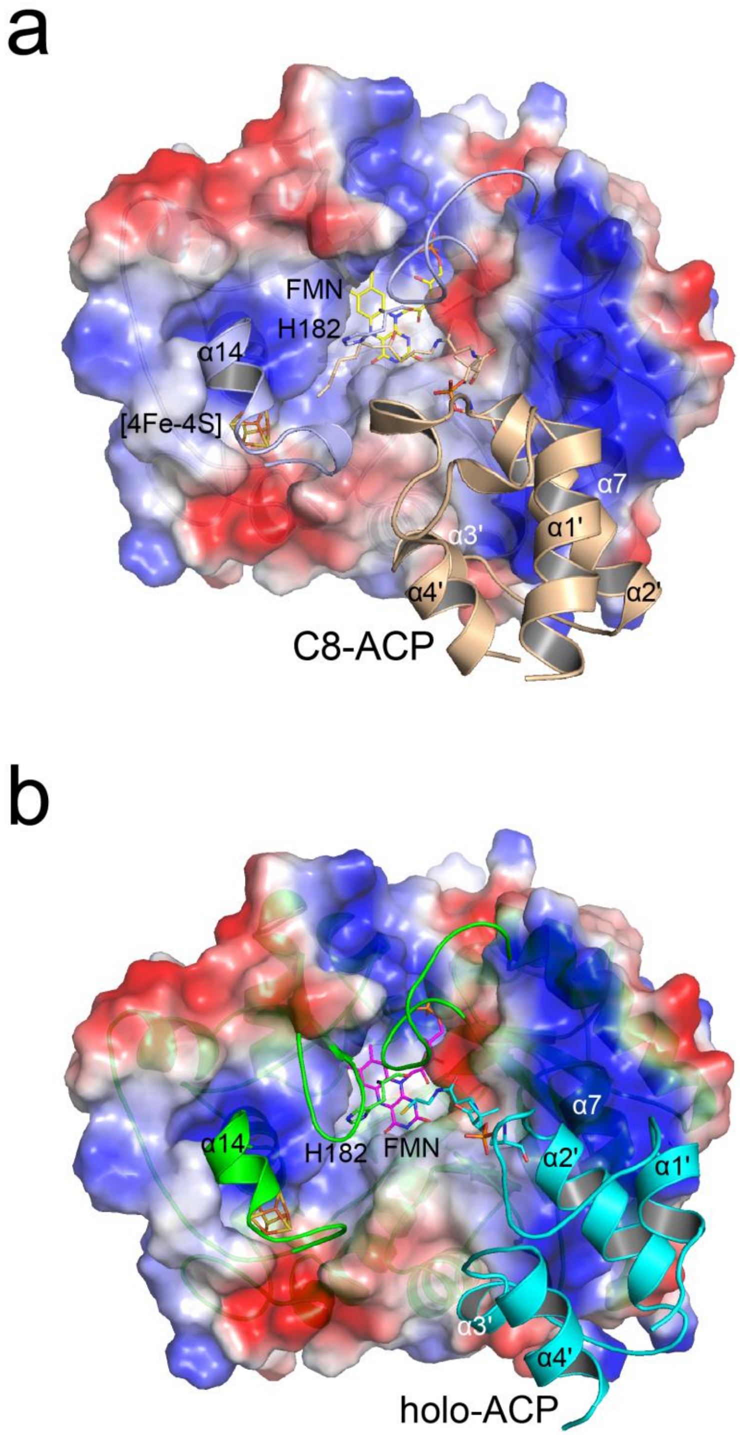

Supplementary Figure 20. ACP binding to FabX. The electrostatic surface of FabX (light blue in a, and green in b) bound by octanoyl-ACP (wheat) (a) or holo-ACP (cyan) (b). Half of the active site tunnel inner surface and the secondary structures of [4Fe-4S] cluster are shown. The FMN cofactor and H182 were also shown in sticks and labeled.

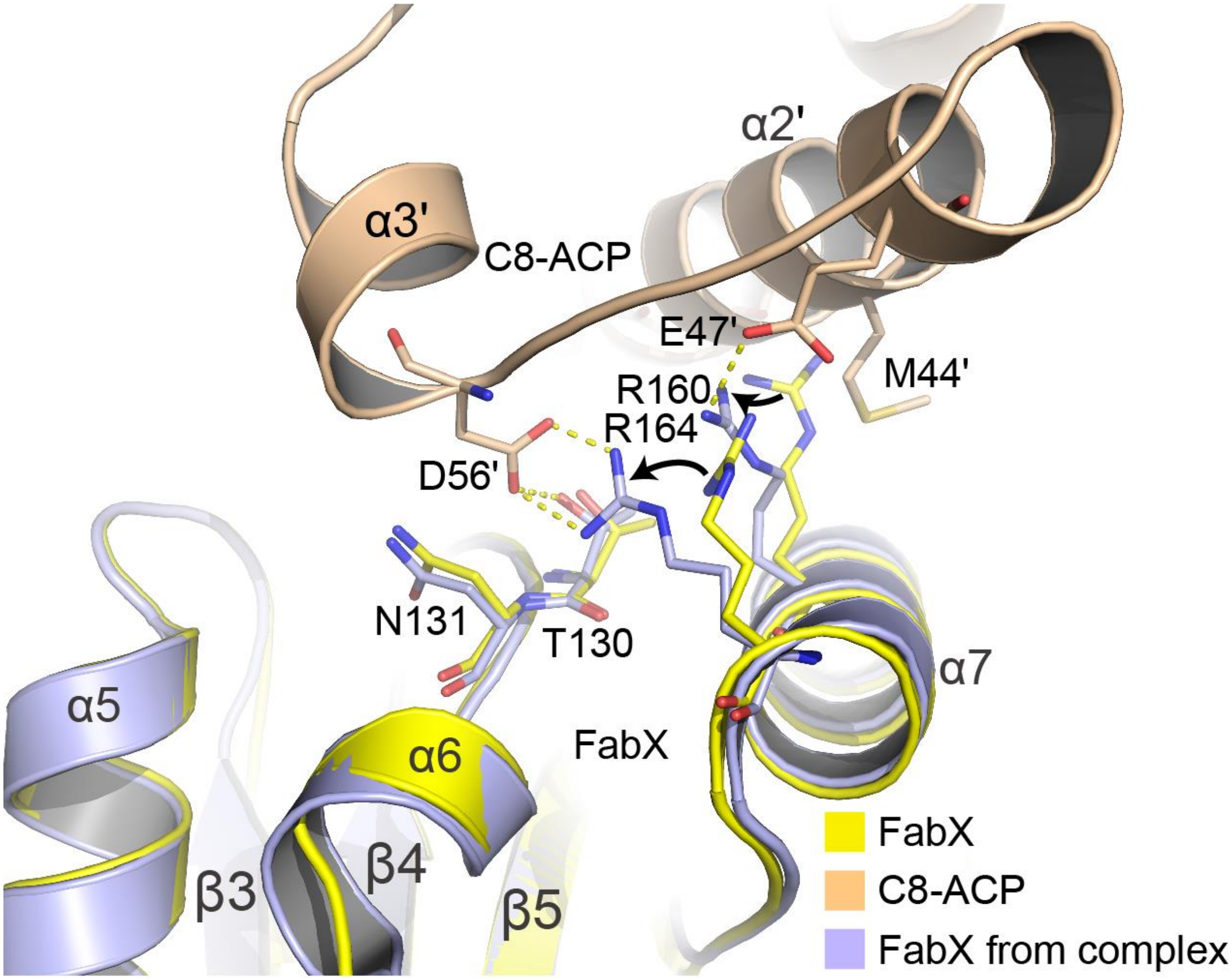

Supplementary Figure 21. Superposition of FabX α7 helix (yellow) and from the FabX-holo-ACP complex (wheat/light blue). The black arrows indicate the rotation directions of the sidechains of residues upon ACP's binding. The yellow dashes indicate the hydrogen bonding between FabX and ACP.

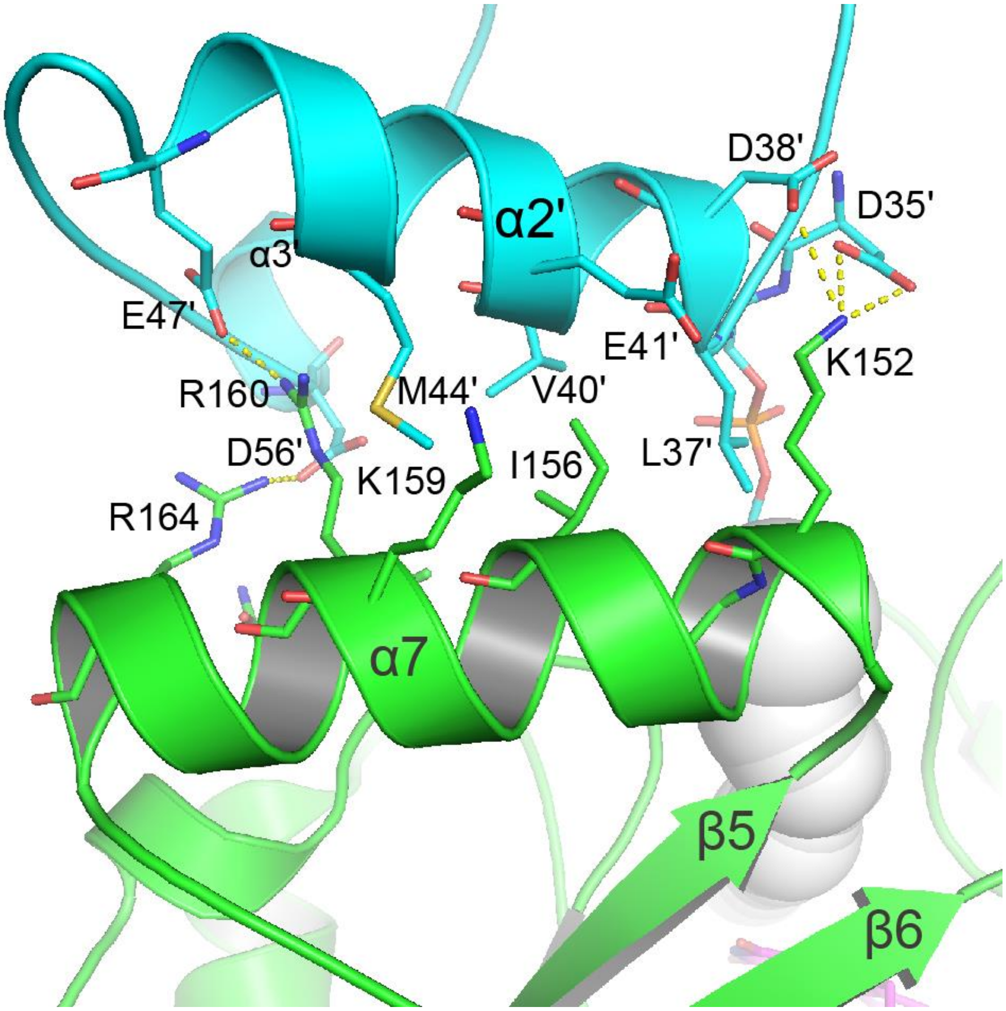

Supplementary Figure 22. Interactions between holo-ACP (cyan) and FabX (green). Residues involved in the binding are shown in sticks and labeled. The primes indicate the residues comes from ACP. Yellow dashes indicate hydrogen bonds between FabX and ACP residues.

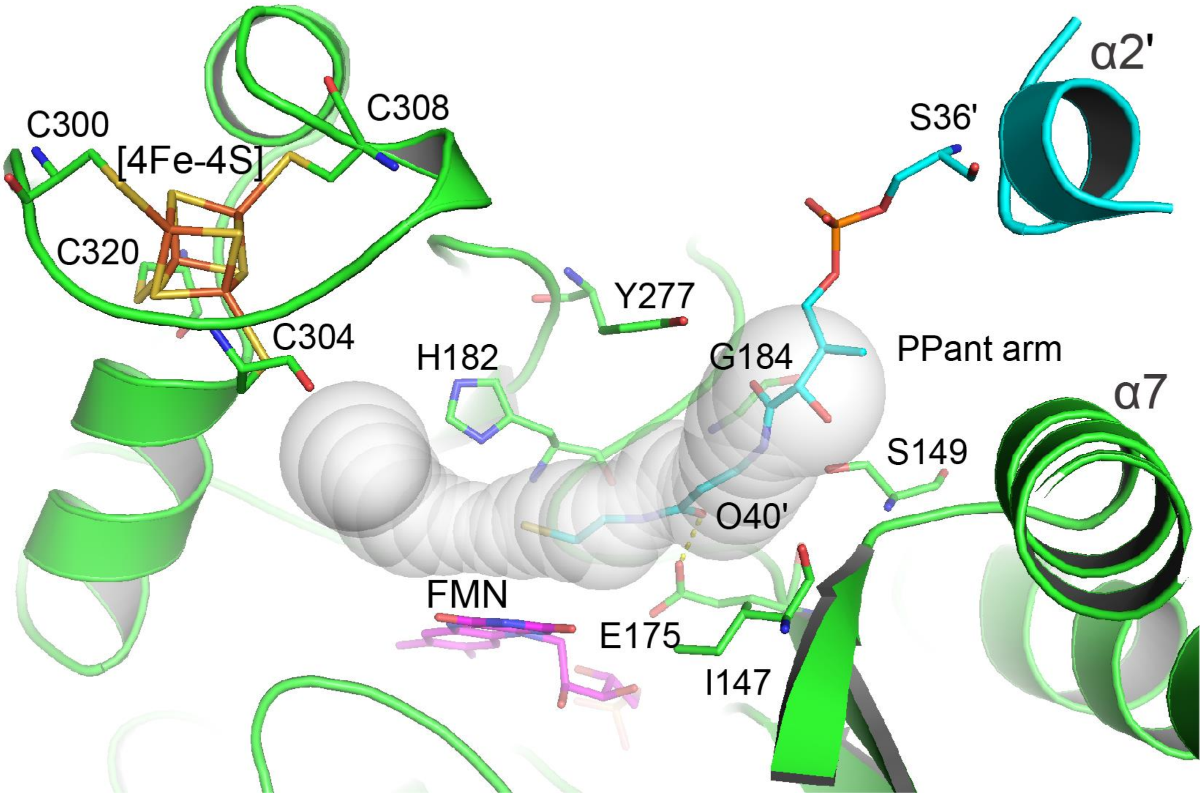

Supplementary Figure 23. Interactions between the PPant prosthetic group of holo-ACP and FabX inside the active site tunnel. Residues that involved in the interactions are shown in sticks and labeled.

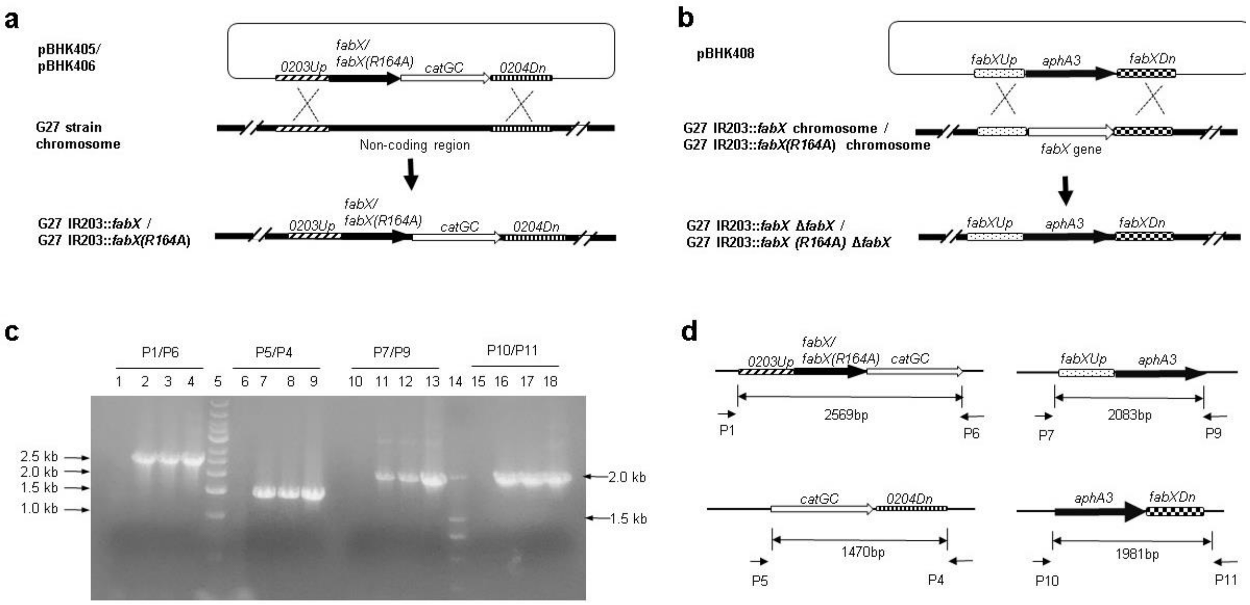

Supplementary Figure 24. Construction and PCR confirmation of the strains BHKS487 (G27 IR203::*fabX* Δ*fabX*) and BHKS488 (G27 IR203::*fabX R164A* Δ*fabX*). (a) Recombination events required to generate the two strains using an improved pIR203C04 complementation system by natural transformation. (b) Recombination events required to generate the *fabX* knockout strains using chromosomal complementation system by natural transformation. (c)(d) PCR confirmation of the constructed strains. As shown in (c), expected product sizes using the primer pairs P1/P6 and P5/P8 for *fabX*/*fabX R164A* insertion into the intergenic region are 2569 and 1470 bp, whereas the expected product size using the primer pair P9/P12 and P11/P14 for *fabX* knockout are 2083 and 1981 bp, respectively. From left to right, the temples (lane1-4 and lane 6-9) used in the PCR reactions are the genomic DNAs of the strain G27, BHKS487, BHKS488, and the plasmid DNA of pBHKP405/406, respectively, while the temples (lane10-13 and lane 15-18) are the genomic DNAs of the strain G27, BHKS487, BHKS488, and the plasmid DNA of pBHKP408, respectively. The experiment was repeated twice independently with similar results. Source data are provided as a Source Data file.

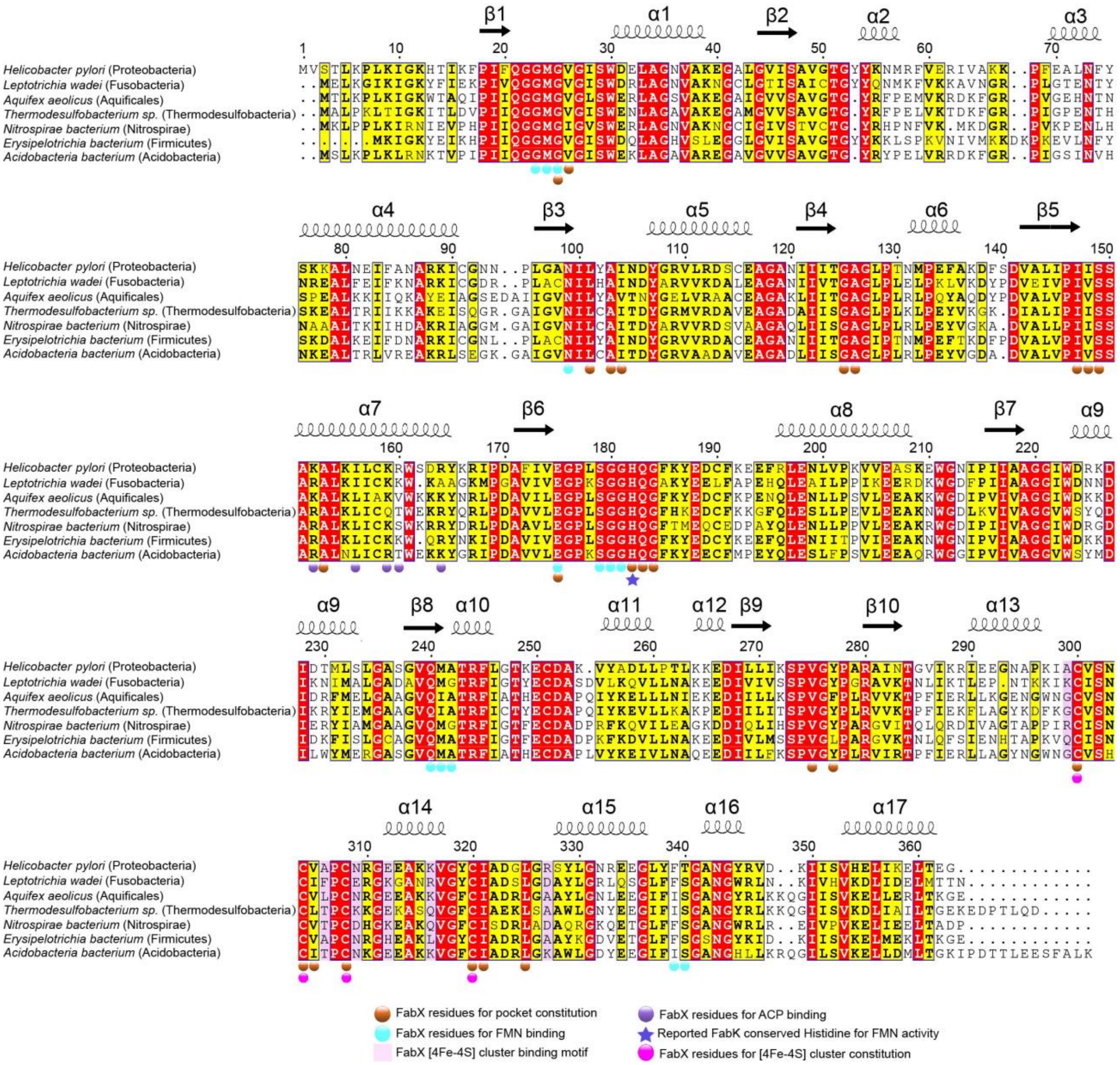

Supplementary Figure 25. Multiple sequence alignments of FabX and the homologs. The secondary structure of FabX is given. Identical and similar residues are shaded in red and yellow respectively. The key active residues of FabK arelabeled with a blue asterisk.

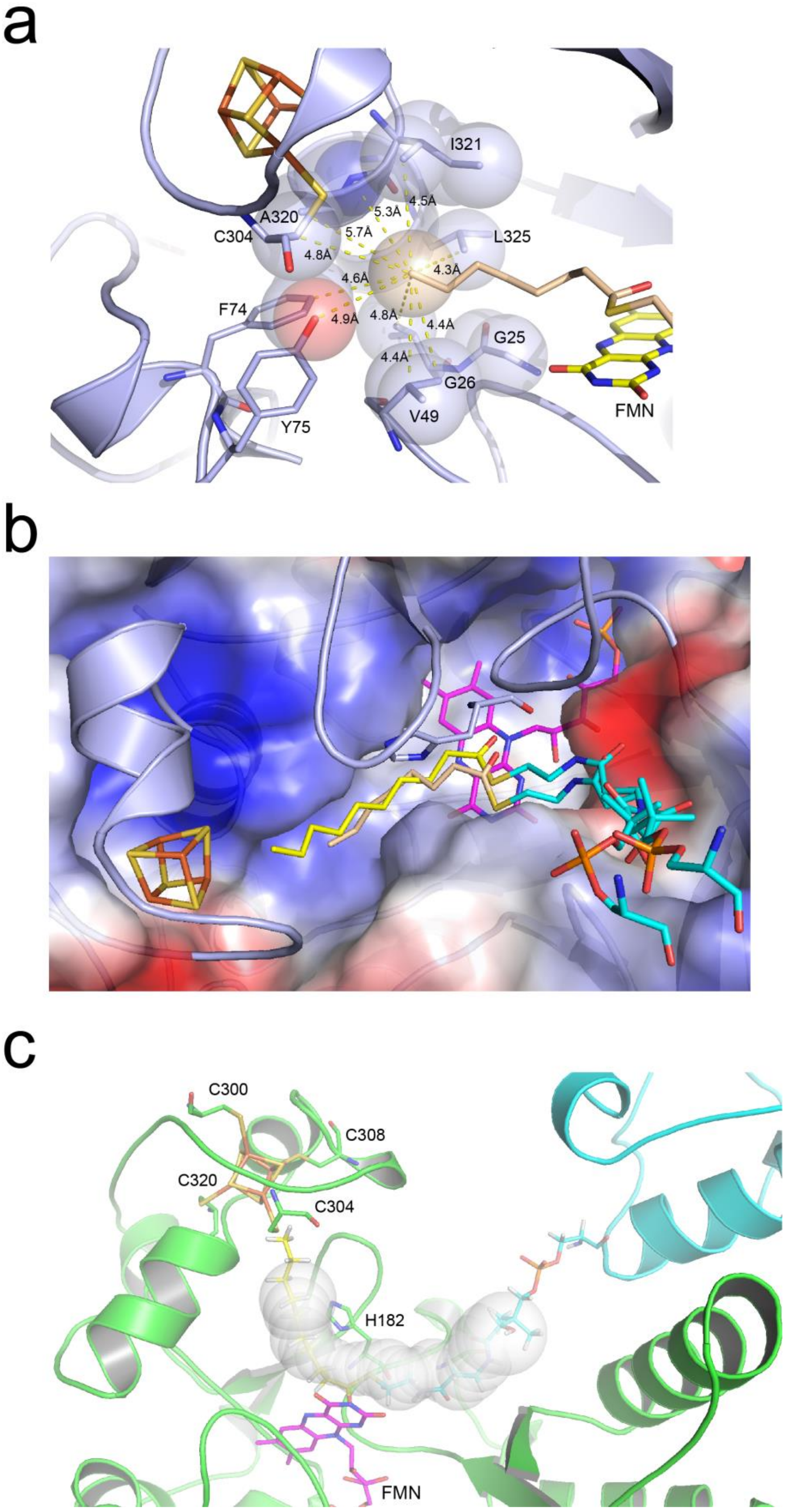
a
I321
4.5Å
5.3Å
A320
5.7Å
L325
C304
4.8Å
4.3Å
4.6Å
F74
4.9Å
4.8Å
4.4Å
G25
4.4Å
G26
FMN
V49
Y75
b
c
C300
C308
C320
C304
H182
FMN

Supplementary Figure 26. Interactions of the terminal methyl group of the octanoyl-group or docked o decanoyl-group in the active tunnel. (a) The octanoyl-chain carried by ACP was shown in wheat sticks and FabX is shown in light blue. The terminal methyl group carbon of the acyl chain and the atoms from FabX residues close to the terminal methyl group are shown as transparent spheres. The FMN cofactor, [4Fe-4S] cluster and residues from FabX involved in the interactions with the acyl chain of the substrate are shown in yellow, orange and light blue sticks. The yellow dashes indicate the distances between the terminal methyl group and the FabX residue atoms. (b) Superposition of FabX-octanoyl-ACP structure with the docked FabX-decanoyl-ACP complex. (c) The decanoyl group (yellow) has close crashes with the [4Fe-4S] cluster.

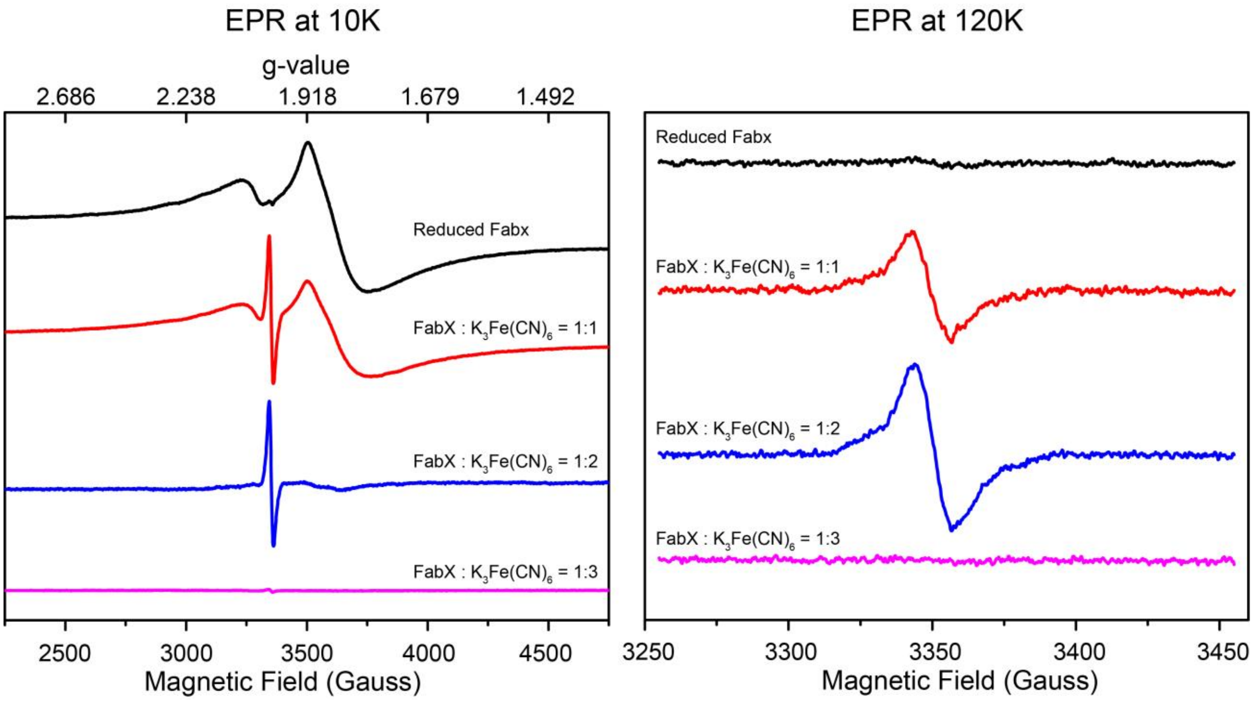

Supplementary Figure 27. Oxidative titration of reduced FabX, monitored by EPR. Different molar equivalents (with respect to protein) of ferricyanide($K_3Fe(CN)_6$) were titrated to dithionite-reduced FabX and EPR spectra were collected using same instrumental parameters. Source data are provided as a Source Data file.

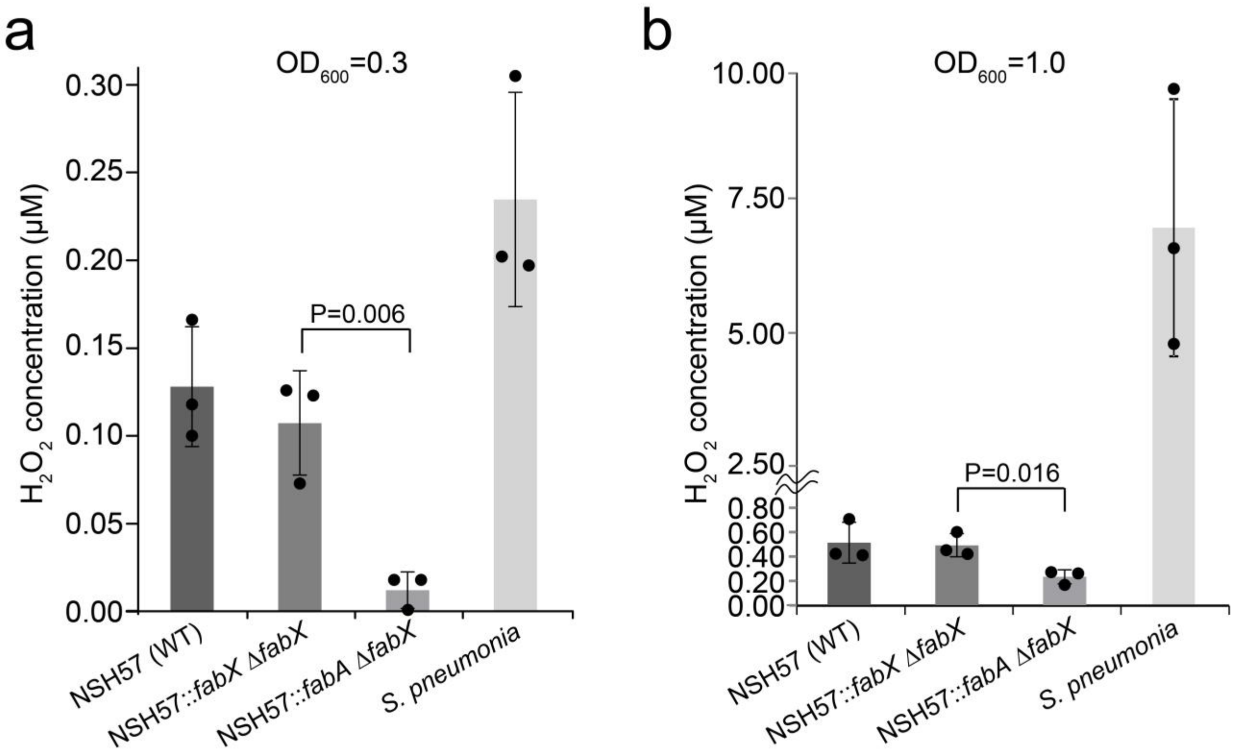

Supplementary Figure 28. H_2O_2 released by *H. pylori* and *S. pneumonia* strains. The strains NSH57, BHKS551 (NSH57 IR0203::*fabA* Δ*fabX*), BHKS568 (NSH57 IR0203::*fabX* Δ*fabX*), and *S. pneumonia* ATCC 49619 were cultured to an OD_{600} of 0.3 (a) and 1.0 (b), respectively. The culture supernatants were collected and directly subjected to H_2O_2 quantification. Data represent the mean (± SD) of three independent experiments with statistical analyses performed by Student's unpaired two-sided t test. Source data are provided as a Source Data file.

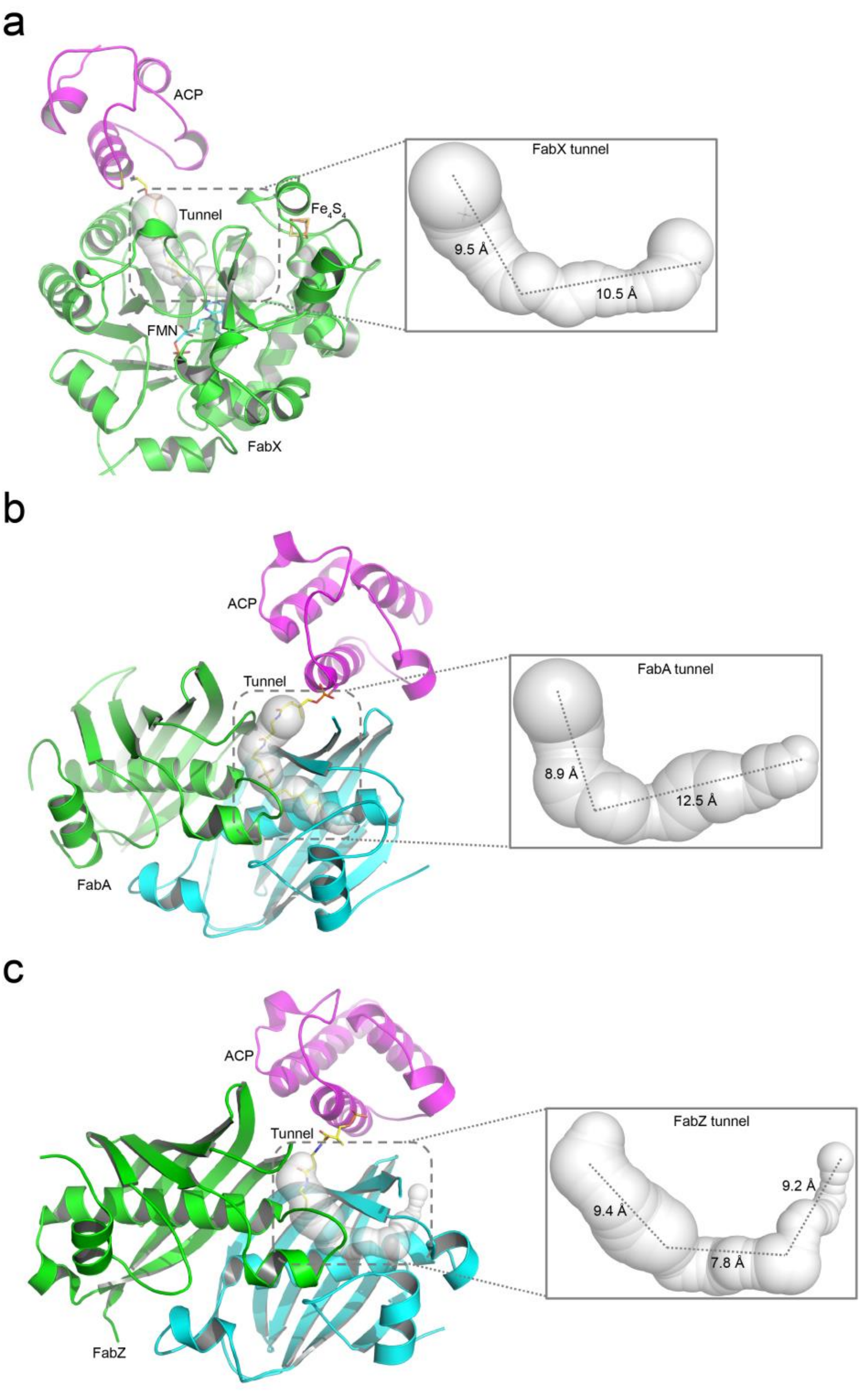

00

Supplementary Figure 29. The active site tunnels of FabX (a), FabA (b) and FabZ (c). FabX and ACP are shown in green and magenta cartoons. FabA (PDB code: 4KEH) and FabZ (PDB code: 4ZJB) dimers are shown in green/cyan cartoons. The white surface of the enzymatic tunnels is shown, and the lengths of the tunnels are shown and labeled.

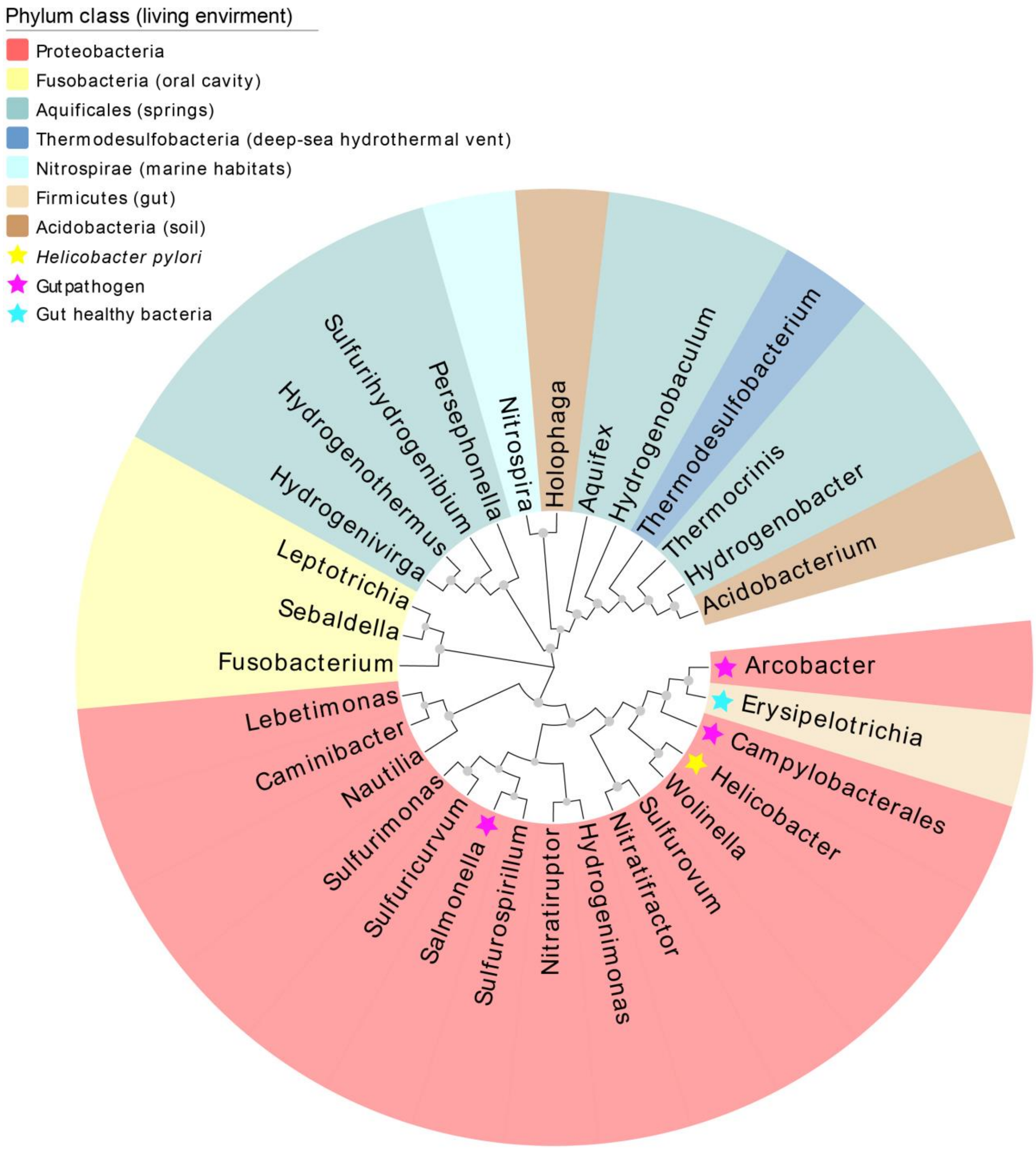

Supplementary Figure 30. Phylogenetic tree of bacteria genera that encode FabX homolog genes. The bacteria that encode FabX homolog (identity higher than 50%, 1650 species in total) belong to 31 genera in 7 phyla. The phyla are highlighted in different colors, including Proteobacteria, Fusobacteria, Aquificales, Thermodesulfobacteria, Nitrospirae, Firmicutes, and Acidobacteria. Yellow, magenta and cyan stars indicate genus *H. pylori*, gut pathogenic and healthy gut bacteria. Grey circles indicate the similarity with FabX.

Supplementary References

1. Baldwin, D. N. *et al*. Identification of *Helicobacter pylori* genes that contribute to stomach colonization. *Infect Immun*. **75**, 1005–1016 (2007).
2. Zhang, L. *et al.* Crystal structure of FabZ-ACP complex reveals a dynamic seesaw-like catalytic mechanism of dehydratase in fatty acid biosynthesis. *Cell Res.* **26**, 1330–1344 (2016).
3. Jiang, Y., Chan, C. H. & Cronan, J. E. The soluble acyl-acyl carrier protein synthetase of *Vibrio harveyi B392* is a member of the medium chain acyl-CoA synthetase family. *Biochemistry* **45**, 10008–10019 (2006).
4. Jiang, X. *et al*. The cyclopropane fatty acid synthase mediates antibiotic resistance and gastric colonization of *Helicobacter pylori*. *J. Bacteriol.* **201**, e00374-19 (2019).
5. Post-Beittenmiller, D., Jaworski, J. G. & Ohlrogge, J. B. In vivo pools of free and acylated acyl carrier proteins in spinach. Evidence for sites of regulation of fatty acid biosynthesis. *J. Biol. Chem.* **266**, 1858–1865 (1991).
6. Chovancová, E. *et al*. CAVER 3.0: A Tool for the Analysis of Transport Pathways in Dynamic Protein Structures. *PLOS Comput. Biol.* **8**, e1002708-e1002719 (2012).
7. White, M. D. *et al.* UbiX is a flavin prenyltransferase required for bacterial ubiquinone biosynthesis. *Nature* **522**, 502–506 (2015).
8. Willistein, M. *et al.* Low potential enzymatic hydride transfer via highly cooperative and inversely functionalized flavin cofactors. *Nat. Commun.* **10**, 2074–2083 (2019).
